# Population analysis of retrotransposons in giraffe genomes supports RTE decline and widespread LINE1 activity in Giraffidae

**DOI:** 10.1186/s13100-021-00254-y

**Published:** 2021-11-26

**Authors:** Malte Petersen, Sven Winter, Raphael Coimbra, Menno J. de Jong, Vladimir V. Kapitonov, Maria A. Nilsson

**Affiliations:** 1 Max Planck Institute of Immunobiology and Epigenetics, Stübeweg 51, 79108 Freiburg, Germany; 2 Senckenberg Biodiversity and Climate Research Centre, Senckenberganlage 25, 60325 Frankfurt am Main, Germany; 3 Institute for Ecology, Evolution and Diversity, Goethe University, Max-von-Laue-Straße 13, 60438 Frankfurt am Main, Germany; 4 LOEWE Centre for Translational Biodiversity Genomics (LOEWE-TBG), Senckenberganlage 25, 60325 Frankfurt am Main, Germany

**Keywords:** Giraffe, Ruminantia, Structural variation, Genome, Transposable elements, TE, Non-LTR retrotransposons, LINE, SINE, LINE1/L1, RTE, BovB

## Abstract

**Background:**

The majority of structural variation in genomes is caused by insertions of transposable elements (TEs). In mammalian genomes, the main TE fraction is made up of autonomous and non-autonomous non-LTR retrotransposons commonly known as LINEs and SINEs (Long and Short Interspersed Nuclear Elements). Here we present one of the first population-level analysis of TE insertions in a non-model organism, the giraffe. Giraffes are ruminant artiodactyls, one of the few mammalian groups with genomes that are colonized by putatively active LINEs of two different clades of non-LTR retrotransposons, namely the LINE1 and RTE/BovB LINEs as well as their associated SINEs. We analyzed TE insertions of both types, and their associated SINEs in three giraffe genome assemblies, as well as across a population level sampling of 48 individuals covering all extant giraffe species.

**Results:**

The comparative genome screen identified 139,525 recent LINE1 and RTE insertions in the sampled giraffe population. The analysis revealed a drastically reduced RTE activity in giraffes, whereas LINE1 is still actively propagating in the genomes of extant (sub)-species. In concert with the extremely low activity of the giraffe RTE, we also found that RTE-dependent SINEs, namely Bov-tA and Bov-A2, have been virtually immobile in the last 2 million years. Despite the high current activity of the giraffe LINE1, we did not find evidence for the presence of currently active LINE1-dependent SINEs. TE insertion heterozygosity rates differ among the different (sub)-species, likely due to divergent population histories.

**Conclusions:**

The horizontally transferred RTE/BovB and its derived SINEs appear to be close to inactivation and subsequent extinction in the genomes of extant giraffe species. This is the first time that the decline of a TE family has been meticulously analyzed from a population genetics perspective. Our study shows how detailed information about past and present TE activity can be obtained by analyzing large-scale population-level genomic data sets.

**Supplementary Information:**

The online version contains supplementary material available at 10.1186/s13100-021-00254-y.

## Background

Transposable elements (TEs) constitute a significant fraction ranging from 30 to 52% in the genome assemblies of most mammals, but even higher amounts up to 69% have been suggested [1–3]. The main fraction of TEs in mammalian genomes is formed by autonomous and non-autonomous retrotransposons without long terminal repeats (LTR), commonly known as LINEs and SINEs (Long and Short Interspersed Nuclear Elements). The autonomous LINEs are about 3-10 kilobase pairs (kb) long and propagate copies of themselves and associated non-autonomous LINEs and SINEs [4]. SINE elements are comparably short, 100-250 base pairs (bp), and are often order-specific [5]. Novel non-autonomous SINEs have emerged in different mammalian orders [2], in contrast to autonomous LINEs, which can be transferred both vertically or horizontally for considerable evolutionary time. Mammalian genomes are characterized by a high abundance of one LINE, the so-called L1/LINE1 [4]. LINE1 has been transferred vertically (parent-offspring) among mammals for at least 167 million years (Myr) [6]. LINE1 is most often vertically transferred, whereas other LINEs, such as RTE/BovB, are frequently involved in horizontal transfers between distantly related groups using intermediate hosts [7, 8]. There are several mechanisms and modes of horizontal transfer which are dependent on the TE type (e.g., [9, 10]) before it can enter the germline and successfully expand in genomes over evolutionary time.

A particularly well-suited mammalian suborder to study TE-activity is the Ruminantia. This clade is one of the few extant placental mammalian groups that have potentially active LINEs from not one but two different clades of non-LTR retrotransposons. An ancient horizontal transfer from an unknown host introduced RTE/BovB into the ancestral ruminant artiodactyl genome around 50 million years ago (Mya) [7, 8]. Over time, the horizontally transferred RTE expanded in copy number and currently makes up around 25% of the ruminant genomes [11]. The horizontal transfer event altered the ancestral ruminant genome to harbor two retrotranspositionally active LINE types instead of one, as is the case in most other extant placental mammals. RTE and LINE1 differ structurally: RTE encodes one open-reading frame (ORF), is ~ 4 kb long, and has a microsatellite sequence at its 3⁠’ end, while LINE1 encodes two ORFs, is 6-8 kb long, and has a poly-A tail at the 3⁠’ end [4, 12, 13].

The clade Ruminantia consists of five families, Bovidae (cattle, sheep, goats, and relatives), Cervidae (deer and relatives), Giraffidae (giraffes), Antilocapridae (pronghorn antelopes), and Moschidae (musk deer) that evolved after their split from Cetacea (whales) around 50 Mya [14]. Giraffidae is one of the taxonomically smaller ruminant families, as it only consists of two extant genera, the okapi (*Okapia johnstoni*) and the giraffe (*Giraffa*). Giraffidae have many morphological and physiological features that make them interesting from a genomic perspective [11, 15, 16]. Genome assemblies of two of the four giraffe species have been published. These include the Masai giraffe (*G. tippelskirchi*) [15, 17] as well as the northern giraffe (*G. camelopardalis*), (subspecies Kordofan (*G. c. antiquorum*) [18] and Nubian (*G. c. camelopardalis*) [16]). To date, all sequenced and assembled ruminant genomes have a similar TE composition with a high percentage of RTE copies [11]. However, it is unclear what type of TEs are still mobile in the giraffe genome, i.e., are currently retrotransposing. Analyses of genome assemblies yield important clues to TE activity; however, only analyses across populations and species provide evidence of which TEs propagate and at what rates. Therefore, there exists a clear need for TE analyses using population-genomic datasets.

Here we perform the first population-level screen of active TEs in Ruminantia, using Giraffidae as a model group. Despite the availability of several computational tools that can identify TE polymorphisms from short sequencing read data, [19–21] large-scale screens at the population level have almost exclusively been applied for primates [20, 22–26] and rarely to non-model organisms [27–29]. We took advantage of a large population data set of giraffe with multiple individuals from all species and subspecies [18] to gain a deeper understanding of the ongoing TE activity in one of the few extant placental mammalian groups that have two potentially active LINEs from different clades of non-LTR retrotransposons. We use the giraffe population-genomic dataset to show how the analysis of TEs can be used to re-evaluate single nucleotide polymorphism based population-genetic inferences and elucidate recent transposon dynamics.

Our results indicate a significant difference in the very recent or current, ongoing retrotransposition activity of the two LINEs. It appears that the giraffe RTEs are on the road to complete inactivation and subsequent extinction.

## Results

### TE content of the giraffe genome

**Fig. 1:**
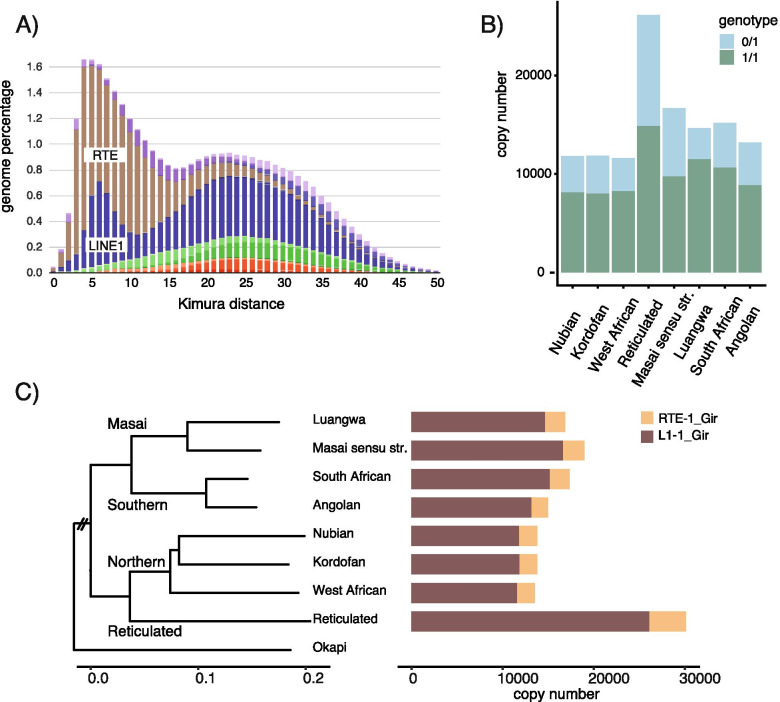
LINE1 and RTE have different activity in giraffe genomes. **A** Repeat Landscape of the Northern Kordofan giraffe genome showing the distribution of LINE1 (dark blue) and RTE (brown) genomic percentage across the Kimura (K2P) distance to the TE consensus sequence. DNA transposons are orange, and LTRs are green. **B** Heterozygous (0/1, blue) and homozygous (1/1, green) L1-1_Gir insertions per subspecies. **C** Number of LINE1 and RTE insertions per subspecies next to the giraffe phylogeny. The giraffe species names are indicated on the internal branches of the phylogeny

We annotated repeats in the Kordofan giraffe assembly using a species-specific repeat library curated to include giraffe-specific LINE1 and RTE consensus sequences. Repeats cover 44.6% of the Kordofan giraffe genome (Table S1). The majority of the repeats in the Kordofan giraffe genome are LINEs (30.5% of the genome), while SINEs cover 3.7% of the genome. The repeat landscape (Fig. 1A), which plots TE content against sequence divergence from the consensus sequence, indicates a recent decline in transposition activity of RTE and LINE1 elements. We derived the two autonomous giraffe-specific consensus sequences RTE-1_Gir (3872 bp) and L1-1_Gir (7997 bp) from the Kordofan giraffe assembly by using either a full-length cattle (*Bos taurus*) LINE1 or searching for coding ORF2s (Fig. S1, Additional file 2) with two different approaches (see Methods). We discovered two additional versions of L1-1_Gir: L1-1A_Gir and L1-1B_Gir. These differ from L1-1_Gir in large deletions in the 5’UTR, while otherwise having a similarity of 99.7% (Fig. S1).

### Comparative analysis of SINE activity in giraffe

The vast majority of SINEs detected in the giraffe genome are insertions belonging to old ruminant SINE families such as Bov-A2 and Bov-tA. There are no novel giraffe-specific SINE families. Active SINEs are distinguished by copies with 100% sequence identity. To examine which giraffe SINEs are currently active, we conducted a large-scale clustering of SINE copies with restrictive clustering parameters (100% identity and at least 98% sequence coverage). Fragmented SINE copies (shorter than the consensus length) were removed from the analysis. Only two families of SINEs, Bov-tA and Bov-A2, form clusters composed of 100% identical copies (Figs. S1 and S2, Additional file 3). However, high similarity in the flanking regions surrounding the SINEs revealed that many of these high-identity clusters result from recent segmental duplication and not retrotransposition. Segmental duplications result in pseudoclusters where the SINE and the flanking regions have 100% sequence identity between copies. We find 381,457 copies of Bov-tA in the giraffe genome that fit our stringent criteria, and from these, 23 clusters are composed of identical copies (Fig. S2A). The largest cluster derived from retrotransposition contains 11 copies. There are 952 additional clusters that each contain two identical copies, resulting from segmental duplications. Similarly, we also find 73,115 Bov-A2 copies suitable for clustering analysis, which includes 85 clusters composed of identical copies. The largest three clusters are composed of 15, 14, and 13 copies derived from recent retrotransposition. Two hundred two-copy clusters originate from segmental duplications (Fig. S2B). To understand whether the high rate of recent segmental duplications containing Bov-tA and Bov-A2 copies is giraffe-specific, we also analyzed the cattle genome assembly. Here, we use 279,199 copies of Bov-tA and 149,118 copies of Bov-A2. Only one Bov-A2 cluster and no Bov-A2 clusters are composed of two identical copies (Fig. S2C, D). This shows the absence of ongoing retrotransposition of Bov-tA and Bov-A2 in the cattle genome. Similar to giraffe, we find a high incidence of segmental duplications (Fig. S2C, D). We screened the giraffe and okapi genome assemblies for the identical Bov-tA and Bov-A2 copies to estimate the time point of insertion by analyzing the flanking sequence and detecting the presence and or absence of the insertion in the different genome assemblies. Of the 42 identical Bov-A2 copies, we find three copies in the giraffe genome and none in the okapi genome, indicating that their insertion occurred after the most recent common ancestor (MRCA) of the extant giraffe species. Of the 81 identical Bov-tA copies, we find only eight copies in the giraffe genome, suggesting they were inserted after the MRCA of the extant giraffe species. The remaining identical Bov-tA and Bov-A2 copies were also observed in the okapi genome; therefore, they were inserted prior to the radiation of the extant giraffe species.

### LINE1 and RTE polymorphic insertions across giraffe species

After a cascading filter (see Methods), 139,525 TE insertions from 48 giraffe individuals remained for analysis (Table S2). In total, 121,237 (86.9%) insertions are LINE1 and 18,288 (13.1%) RTE (Table 1). We estimated the genotype (homozygosity/heterozygosity) based on the coverage of the insertion. The average ratio of heterozygous insertions is significantly different between RTE (23.0%) and LINE1 (33.3%) (ANOVA, F = 47.3, *p* = 6.7e-10). Also, the heterozygosity ratio differs between species and subspecies (Table S3, Fig. 1B). An ANOVA with phylogenetic independent contrasts (PIC [30];) identified a non-significant difference in the ratio of heterozygous to homozygous insertions between the four giraffe species and subspecies for both LINE1 and RTE. Among the giraffe subspecies, the Luangwa giraffe (*G. t. thornicrofti*), a subspecies of the Masai giraffe, has the lowest overall heterozygosity, while the Kordofan giraffe, a northern giraffe subspecies, has the highest. Mapping the number of insertions to the giraffe phylogeny revealed excess insertions only on short branches (Figs. S6 and S7).

**Table 1:** Numbers of species- and subspecies-specific insertions of L1-1_Gir and RTE-1_Gir retrotransposons in giraffe genomes

Species	L1-1_Gir	RTE-1_Gir	Combined
Northern giraffe	35,435	5821	41,256
-Kordofan	11,872	1947	13,819
-Nubian	11,844	1948	13,792
-West African	11,629	1926	13,555
Reticulated giraffe	26,141	4009	30,150
Southern giraffe	28,404	3943	32,347
-Angolan	13,201	1777	14,978
-South African	15,203	2166	17,369
Masai giraffe sensu *lato*	31,347	4515	35,862
-Masai sensu stricto	16,677	2310	18,987
-Luangwa	14,670	2205	16,875
Total	121,237	18,288	139,525

Northern giraffe (*G. camelopardalis*), Kordofan giraffe (*G. c. antiquorum*), Nubian giraffe (*G. c. camelopardalis*), West African giraffe (*G. c. peralta*); Reticulated giraffe (*G. reticulata*); Southern giraffe (*G. giraffa*), Angolan giraffe (*G. g. angolensis*), South African giraffe (*G. g. giraffa*); Masai giraffe sensu *lato* (*G. tippelskirchi*), Masai giraffe sensu stricto (*G. t. tippelskirchi*), Luangwa giraffe (*G. t. thornicrofti*)

As expected, the length distribution of L1-1_Gir shows that the majority of the insertions are shortened copies due to the frequent 5′-truncation of LINE1 during insertion (Fig. S3). There is a marked peak of full-length copies around 8000 bp, which is the LINE1 consensus sequence length. In total, 6438 L1-1_Gir recent polymorphic insertions are longer than 7980 bp (Table S4), which is 5.3% of the total LINE1 insertions. Among the RTE-1_Gir insertions, we find few full-length copies across the giraffe species. Only 59 RTE-1_Gir copies are longer than 3850 bp (Table S4), which is 0.32% of the total number of all RTE insertions.

To further investigate the near lack of full-length RTE-1_Gir polymorphisms, we screened two Masai and one northern giraffe reference genome assemblies. The two Masai giraffe genome assemblies MA1 and OR1865, use data from the same individual but were assembled using different approaches [11, 17]. Using the 3132 bp ORF from the RTE-1_Gir consensus sequence as a query, we identified ORFs in the three assemblies. We find 3049 full-length ORFs in the northern giraffe assembly, while the two assemblies from the same Masai individual differ more than twofold in number of ORFs (OR1865: 1126 copies; MA1: 2547 copies) (Table S5). The threefold difference in RTE ORF numbers between the northern giraffe assembly and the Masai giraffe assembly (OR1865) is due to numerous long runs of Ns present inside RTE elements in the assembled genomic copies of OR1865 that interrupt ORFs and hamper identification. Among the identified full-length ORF copies, only a very limited number (9: Kordofan; 5: MA1; 0: OR1865) are intact ORFs coding for the 1044 amino acid protein containing the endonuclease and reverse transcriptase catalytic domains.

To trace the evolution of these 14 intact RTE copies identified in the northern and Masai giraffe genomes, we screened for their orthologs using the 280 bp flanking sequences in all Bovidae sequences in GenBank using BLASTN to detect their presence and/or absence to show that most of these copies were retrotransposed in Giraffidae (Fig. S4).

### Phylogenetic distribution of recent LINE1 and RTE insertions

**Fig. 2:**
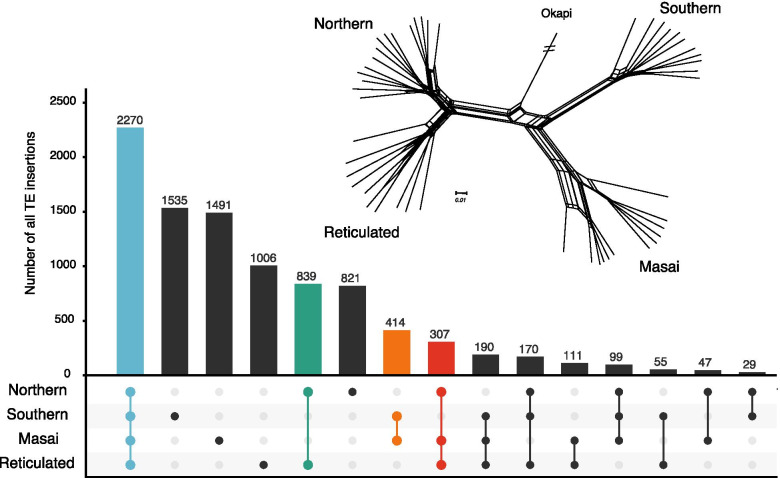
Phylogenetic incongruence of TE insertions across the giraffe species complex. NeighborNet network and UpSet plot showing the supporting TE insertions for different nodes in the giraffe data set. 2270 insertions (blue) support the grouping of the four species. Each species is supported by between 1535 (southern) to 821 (northern) unique insertions. 839 insertions (green) support northern and reticulated giraffe and 414 insertions (orange) support Masai and southern giraffe. 307 insertions (red) support the clustering of northern, Masai, and reticulated giraffe. The branch of the outgroup okapi has been shortened. See Fig. S5 for the NeighborNet tree with individual names

We compiled a phylogenetic data set of 9382 LINE1 and RTE insertion loci for 48 individuals and an outgroup. The data set includes a total of 8622 parsimony-informative characters, 760 singleton sites, and no constant sites. We used three different tree reconstruction methods with this dataset that all yield four well-defined taxonomic units, equalling the four proposed giraffe species [31]. A single most parsimonious tree was identified with PAUP [32] (tree length 44,323) with a consistency index (CI) of 0.212 and homoplasy index (HI) of 0.788 which indicated the presence of conflicting signals in the data set (Fig. S5A). The parsimony tree supports a phylogeny where the northern and reticulated giraffe are sister groups to the southern and Masai giraffe. The same topology was identified using a Neighbor-Joining approach (Fig. S5C). A phylogenetic network analysis using NeighborNet reconstructed an identical topology but indicated phylogenetic conflict for most of the nodes (Fig. S5B, Fig. 2) as suggested by the high HI/low CI.

An UpSet intersection plot of the phylogenetic data set shows that the strongest signal (838 insertions) in the data set supports a close relationship between northern and reticulated giraffe (Fig. 2). The relationship between Masai and southern giraffe is supported by 414 insertions. However, there are several conflicting insertions. For instance, the support for a sister group position of southern giraffe to northern, reticulated, and Masai giraffe is supported by 307 insertions. One individual, RET3, a known hybrid between northern and reticulated giraffe, was placed at a nested position between the two species. The different lineages are each supported by 1535 (southern), 1491 (Masai), 1005 (reticulated), and 821 (northern) novel and unique insertions, as reflected by the support for four taxonomic units in each of the phylogenetic analyses.

**Fig. 3:**
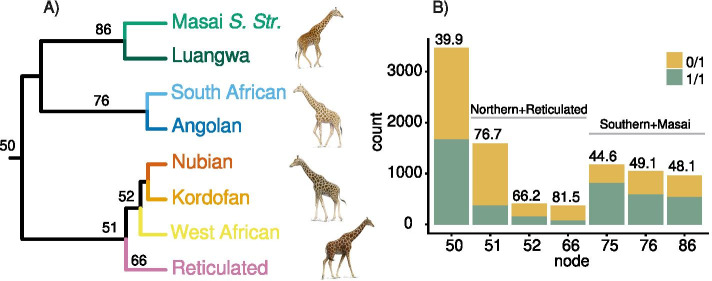
TE heterozygosity differs across giraffe lineages. Higher proportions of heterozygous TE-insertions are observed in the lineages leading to northern and reticulated giraffes compared to lineages leading to southern and Masai giraffe. **A** UPGMA-tree based on a 9.4 K TE-dataset, showing the node numbers referred to in (**B**). **B** Barplot showing the number of homozygous (1/1, green) and heterozygous (0/1, yellow) TE-insertions inferred per node. Values above the bars indicate the percentage of heterozygous insertions (Table S6). Giraffe images by Jón Baldur Hlíðberg

We find that the heterozygosity rate of the TE insertions differs between the giraffe species and the insertion age (Fig. 3). TEs that are inserted in the ancestor to all four giraffe species have a heterozygosity of 39.9% (Fig. 3A, B, Table S6). We also found an accumulation of full-length LINE1 insertions in the Masai and southern giraffe species (Fig. 3B, Tables S4, S7 and S8). Similarly, there is a higher abundance of full-length RTE insertions in Masai and southern giraffe than in reticulated and northern giraffe (Table S4), although at a much lower rate than LINE1.

### Comparison between TE- and SNP-based inferences

**Fig. 4:**
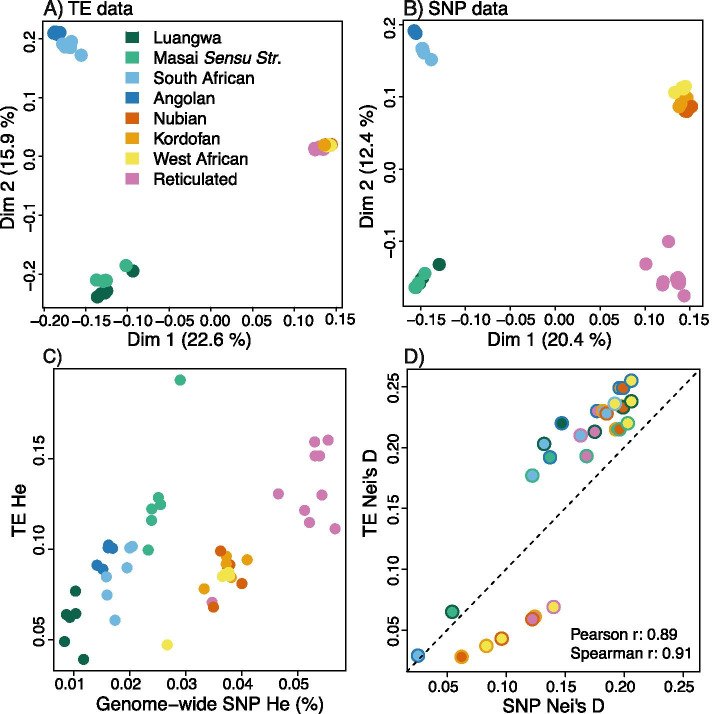
A comparison between population-genetic inferences from TE and SNP data. TE-based population differentiation and genetic diversity estimates are generally in agreement with SNP-based inferences, except for the northern (Nubian, Kordofan and West African) giraffes. **A** Principal component analyses using a 48 K SNP dataset. **B** Idem, using a 9.4 K TE dataset. **C** Scatterplot showing sample specific heterozygosity estimates inferred from a 9.4 K TE dataset (y-axis) against genome-wide heterozygosity estimates (x-axis) reported by Coimbra et al. (2021). **D** Scatterplot showing Nei’s genetic distance estimates between subspecies inferred from a 9.4 K TE dataset (y-axis) against estimates inferred from a 48 K SNP dataset (x-axis). Color-coding follows the phylogeny in Fig. 3

The three TE datasets (LINE, RTE and combined) revealed similar genetic clustering patterns of the giraffe (Fig. S8, B, C, D). We used the combined dataset for the comparison with SNP-based inferences. For the TE dataset as well as the SNP dataset, the first Principal Component Analyses-axis (PCA) separated Masai and southern giraffes from northern and reticulated giraffes (Fig. 4A, B). Furthermore, for both datasets the second PCA-axis separated Masai giraffes from southern giraffes (Fig. 4). However, whereas in the case of the SNP data the second axis also separated northern giraffes from reticulated giraffes, for the TE data this distinction was revealed by the third PCA-axis only (Fig. 4, S8). PCoA-biplots and NJ-phylogenies revealed the same clustering patterns, showing general congruence between the TE and SNP-based inferences, except regarding the genetic distance between northern and reticulated giraffes (Fig. 4, S5 and S9). Population differentiation estimates obtained from the TE dataset correlated strongly with estimates obtained from the SNP dataset. Pearson correlation coefficients equalled 0.89, 0.91 and 0.96 for pairwise population estimates of Nei’s D [33], Wright Fst [34] and Weir and Cockerham Fst [35] respectively (Fig. 4D, Table S9). Deviations were predominantly observed for pairwise comparisons involving northern and reticulated giraffes (Fig. 4D, Table S9). Consistent with the results from clustering analyses, the population differentiation estimates from the TE dataset indicated lower genetic distance between northern and reticulated giraffes than the estimates from the SNP dataset. The correlation between TE heterozygosity (He) and genome-wide heterozygosity (i.e., the proportion of single nucleotide heterozygous sites) depended on the method used to calculate TE heterozygosity. Genome-wide He correlated better with TE He estimates obtained for segregating sites (TE He_seg_) than with TE heterozygosity obtained for segregating and non-segregating sites combined (TE He_all_) (Fig. 4A, Fig. S9). The discrepancy was mainly caused by the northern giraffes, which scored relatively low TE-He_all_ estimates (Fig. 4A). A few individuals showed TE He levels which deviated from population averages (Fig. 4A). Reticulated giraffe ‘ISC01’ and northern giraffe ‘WA746’ scored relatively low levels of TE-He and SNP-He. Masai giraffe ‘MA1’ scored elevated levels of TE-He, but a normal level of SNV-He (Fig. S10 (same as Fig. 4A but with names)).

## Discussion

### Population-level data reveals recent transposon dynamics

The population-level data set of TE-insertions provides insight into the recent dynamics of retrotransposons in giraffe. The analysis of the giraffe genomes revealed large differences in retrotransposition activity of the autonomous retrotransposons LINE1 and RTE. The majority (121,237 insertions, 86.9%) of polymorphic insertions identified among the giraffe species originate from LINE1 retrotransposition. RTE propagates at a much lower rate than LINE1 in giraffe and accounts for around 13.1% (18,288 insertions) of all insertions. Despite the low number of recent insertions, an in-depth in silico screen of the available giraffe assemblies identified several potentially functional autonomous RTE copies. However, a comparative analysis shows that the majority of the coding full-length RTE copies inserted after the split from okapi but before the split to all extant giraffe species, which makes them at least 1–2 Myr old. In addition, the recent polymorphic RTE insertions are shorter than the consensus sequence length, leading to incomplete 5′ UTRs and unlikely to be functional. Detailed analysis of the giraffe RTE-associated SINEs indicates that these are propagating at an extremely low rate. Taken together, the results suggest that RTE has become mostly inactive in the giraffe genome. RTE is a large part of the TE landscape in all ruminant genomes [11, 36, 37]. However, both the giraffe and cattle genome contain only a few potentially active RTE copies [36], suggesting that RTE might have an evolutionary disadvantage over longer evolutionary timescales compared to LINE1. RTE elements have invaded the genome of several mammalian groups (e.g., bats, perissodactyls, afrotherians, monotremes, marsupials) through horizontal transfer [38, 39]. However, in general, only a few remnants of inactive copies are present in the genomes, which suggests that RTE is now prone to extinction, around 50 million years after its introduction into the ruminant genome via horizontal transfer [7, 8]. Furthermore, the congruent observations in both giraffe and cattle genomes indicate that RTE activity is declining in other Ruminantia as well despite their initially strong dispersion.

The dynamics of LINE1 in the giraffe genome are the opposite to RTE, as we found around 121,000 polymorphic insertions and more than 6400 full-length copies. Before the horizontal transfer of RTE, the genomes of ancestral, now extinct, ruminants contained active LINE1 and SINEs (CHR-SINEs) [40, 41]. The ancestral LINE1 propagated SINEs became inactive in Ruminantia at the time of the RTE invasion [40]. The results of our clustering analyses indicate that no new LINE1-propagated SINEs have formed in giraffe. The only current activity of non-LTR retrotransposons is that of autonomous LINE1s. Thus, LINE1 is still actively creating structural variation in giraffe populations by generating new insertions.

LINE1 has been active in mammalian genomes since the split between marsupial and placental mammals around 150–160 Mya [1]. Unlike RTE, LINE1 is less prone to inactivation. However, the genomes of a few mammalian groups contain inactivated LINE1, such as megabats, sigmodontine rodents, among others [42–47]. Thus, during the evolution of Giraffidae, the activity of RTE and RTE-derived SINEs decreased, but LINE1 lingers as the main retrotransposition driver in the giraffe genome.

### Beyond SNPs: TEs as an independent marker to address population-genetic questions

With the advent of next generation sequencing, SNP-markers have become the method of choice for population-genetic inferences. However, genomic data contains other types of polymorphisms which could serve similar purposes. We investigated the feasibility of TEs for population-genetic analyses by reevaluating the population structure and genetic diversity of giraffe populations, a study system which recently has been examined using SNP markers [18]. We find good congruence between the TE-based inferences and SNP-based inferences. The population clustering suggested by the TE dataset generally agrees with the clustering suggested by SNPs. This finding shows that TEs, like SNPs, can serve as a marker in population-genetic studies, and furthermore refutes concerns about the extraction of TE genotypes from short read sequencing data.

The genus *Giraffa* is considered to encompass four species [18, 31, 48]. The species relationship has been explored using different data sets which resulted in a consistent topology where the two species occurring in the northern part of Africa, the northern and reticulated giraffe, are sister species [18, 31, 48]. The relationship between the Masai and the southern giraffe has been more challenging to resolve; however, whole-genome analyses suggest that these are sister species [18]. Extant Masai and southern giraffe occur in eastern and southern Africa, respectively, and are geographically separated from the northern and reticulated giraffe [49]. Our phylogenetic and structure analyses of the LINE1 and RTE insertions agree with the whole genome phylogeny and support the current four species taxonomy proposed by [18, 31, 48]. One difference concerns the genetic distance of populations in northern and eastern Africa: the TE-markers suggest a lower genetic distance between northern and reticulated giraffes than inferred from SNP data.

Our phylogenetic analysis of TE insertions distinguishes the presence of the seven recognized giraffe subspecies. In particular, the clustering of the individuals from the Luangwa valley is well supported. The Luangwa giraffe has the lowest number of heterozygous TEs of the seven subspecies. Currently, only 600 individuals occur in the wild in the Luangwa valley national park. The resulting high rate of inbreeding and possible bottleneck offers an explanation for the high numbers of homozygous TEs and SNPs [18]. The data set included a zoo hybrid between reticulated and northern giraffe. Network analyses of the TE insertions place the hybrid individual nested between the reticulated and northern giraffe, as expected. Thus, there is high congruence between the whole-genome analyses from [18] and our results, which reinforces the confidence in the capability of TE insertion datasets from large scale SV calling approaches to resolve phylogenies [27].

Whole-genome analyses of SNPs and runs of homozygosity (ROH) can reveal past population structure both on a population and species level [50, 51]. Among giraffe, both Masai and southern giraffe have low levels of genomic SNP heterozygosity and longer ROH, which suggests inbreeding [18]. However, both Masai and southern giraffe have large populations with ~ 35,000 (Masai) and ~ 52,000 (southern) individuals in the wild [49]. The opposite is observed for northern and reticulated giraffe, with small populations between 8600 (reticulated) to 4700 (northern) individuals [49] and high genomic heterozygosity as well as short ROHs [18]. The apparent conflicting signals regarding extant population sizes and genetic variability have been difficult to explain for giraffe, but similar inconsistencies have been observed for other animal populations [52].

TE copies insert in the genome in one copy at each locus (heterozygous) and will become homozygous (two copies) over time in the population. TE insertions become fixed faster in small populations and slower in large populations [53], similar to SNPs. The analysis of TE heterozygosity in the giraffe populations reveals a similar pattern to that from the SNP analysis by [18], except regarding the northern giraffes. The arithmetic mean coverage of our genome data set is 19X, which was shown to be optimal to reliably call both TE insertions and genotypes [25, 54]. By analyzing the heterozygosity of older TE insertions at deeper nodes in the phylogeny, we find that the clade-specific heterozygosity patterns have already originated in the MRCA to the species. The TE insertions that integrated into the genome of the MRCA of northern and reticulated giraffe have a heterozygosity of close to 76%. In comparison, in the MRCA of Masai and southern giraffe, the heterozygosity is only 44%. Thus, the ancestral population that gave rise to northern and reticulated giraffe likely had a very large population size, while the ancestral population to Masai and southern giraffe had a much smaller population size. This closely mirrors previous findings on past effective population sizes (Ne) obtained through coalescent modelling, which indicate low N_e_ of Masai and southern giraffe compared to northern and reticulated giraffe [18]. The low N_e_ suggests that TEs became fixed in the population at a higher level than in the northern and reticulated giraffe MRCA. Our analysis shows that past population dynamics in the ancestors of the extant giraffe species have strongly influenced the differences in TE activity and fixation rate of TEs in the four giraffe species.

## Conclusions

Our large-scale population analysis of four giraffe species provides detailed insights into the ongoing activity of TEs in ruminant genomes. The RTE retrotransposition is driven by older master copies that seemingly lost the capability to create new full-length insertions. There is currently extremely low or no associated SINE activity as RTE is unable to retrotranspose SINEs efficiently, nor are there recent LINE1-propagated SINEs. Unless new horizontal transfers of RTEs occur, RTE will likely go extinct in the giraffe lineage. In contrast to RTE, there is ongoing LINE1 retrotransposition activity, which is the main driver of retrotransposition in giraffe genomes. By tracing the pattern of TE activity back in time and across populations, we can better understand the origin of activity and heterozygosity differences between TEs in ruminant genomes and beyond.

## Methods

### De novo repeat library construction

We used RepeatModeler version 2.0.1 [55] with the option ‘-LTRStruct’ to characterize Giraffidae-specific non-LTR TEs in the northern giraffe, subspecies Kordofan (*G. camelopardalis antiquorum*), genome assembly (ASM1828223v1) from [18]. RepeatModeler creates a de novo repeat library for downstream annotation.

### Giraffe-specific RTE and LINE1 consensus sequences

To complement the de novo repeat library by RepeatModeler, we derived RTE and LINE1 giraffe-specific consensus sequences by specifically searching for RTE and LINE1 copies in the Kordofan giraffe genome assembly. We focused on consensus sequences from the youngest and potentially active elements, which are characterized by either one (RTE) or two (LINE1) intact coding ORFs, and intact 5′ UTR and 3′ UTR. To identify full-length insertions, we extracted sequences similar to ORF2 from cattle RTE/BovB and L1-BT (RepBase) from the Kordofan giraffe genome and used MAFFT version 7.475 (parameters L-INS-I) [56] to generate multiple alignments. We included only giraffe sequences whose length differed by less than 100 amino acids from the cattle L1-BT ORF2. We extracted the flanking regions (between 140 bp to 4500 bp depending on TE type and flanking region) from the genome assembly to arrive at full-length sequences of LINE1 and RTE copies. Additionally, we derived consensus sequences from the top 100 giraffe sequences most similar to the canonical cattle BovB and L1-BT sequences. These were identified using BLAST implemented in Censor version 4.2 [57, 58]. We also extracted copies of these TEs in the cattle and okapi genome assemblies (cattle:ARS-UCD1.2/GCA_002263795.2;okapi: ASM166083v1/GCA_001660835.1). We computed multiple DNA sequence alignments from the TE copy sequences with MAFFT version 7.475 (parameters L-INS-I) and edited them manually in SEAVIEW version 5.0.4 [59] to remove truncated copies, remove copies with small indels, and check for the existence of target site duplication (TSD) at the 5′ and 3′ end. We built consensus sequences using the majority rule applied to the modified multiple sequence alignments of TE copies, including a reversal of the ancestral CpG dinucleotides mutated into the TpG and CpA dinucleotides to account for the fast methylation decay. We discarded TE copies created by chromosomal segmental duplications when the identity between the corresponding 350 bp flanking regions was ≥0.98.

### Repeat annotation of the Kordofan giraffe genome

We merged the de novo RepeatModeler library with Cetartiodactyla-specific TEs from RepBase version version 20181026, including the newly generated giraffe-specific LINE1 and RTE consensus sequences to arrive at our final giraffe-specific TE library. We used RepeatMasker version open-4.0.9 [60] to annotate repeats in the Kordofan giraffe genome assembly with this giraffe-specific TE library. Repeat landscapes were created using the RepeatMasker utility scripts.

### Clustering copies of RTE- and LINE1-dependent SINEs

To identify the copies of RTE and LINE1 dependent SINEs, we used the representative set of extracted consensus sequences composed of RTE-1_Gir and RTE-dependent SINEs, including Bov-tA1, Bov-tA2a_Gir, Bov-tA2a1_Gir, Bov-tA2b_Gir, Bov-tA2c_Gir, Bov-tA2d_Gir, Bov-tA2e_Gir, Bov-tA3_Gir, Bov-tA3a_Gir, BTALUL1, Bovc-tA2, Bov-A2_Gir, Bov-A2b_Gir, Bov-A2c_Gir, Bov-A2d_Gir, Bov-tA-monoA_Gir, Bov-tA-monoB_Gir [40, 61, 62] (Fig. S1, Additional file 2) used as a query library with Censor version 4.2. To find active SINEs, we identified clusters composed of identical copies of the RTE-dependent SINE elements. From all identified SINE copies, we extracted DNA sequences of those that were full-length copies of the corresponding consensus sequences. We considered a SINE copy a full-length copy if its termini were truncated by less than 15 bp compared to the corresponding consensus sequence. We clustered all selected full-length copies using MMSEQS2 release 13-45111 [63] in the easy-cluster mode with the parameters: min-seq-id = 1.0, c = 0.98, cov-mode = 0. We used the same approach to identify clusters composed of identical copies of LINE1-dependent SINEs. The corresponding query library of LINE1-dependent SINEs was composed of the CHR-2_Gir, CHR-2_BT, CHR-2A, SINE2-1_BT, SINE2-2_BT and SINE2-3_BT consensus sequences (Fig. S1, Additional file 2).

### TE insertion calling and filtering

We included genomic data of 48 individuals covering all four giraffe species and seven subspecies from [18] for the TE analysis (Table S2). The northern giraffe (*G. camelopardalis*) was represented by 15 individuals, including its three subspecies: the Nubian (*G. c. camelopardalis*), the Kordofan (*G. c. antiquorum*), and the West African giraffe (*G. c. peralta*). The reticulated giraffe (*G. reticulata*) included ten individuals. The Masai giraffe sensu *lato* (*G. tippelskirchi*) was represented by 12 individuals, including its two subspecies: the Luangwa (*G. t. thornicrofti*) and the Masai giraffe sensu stricto (*G. t. tippelskirchi*). Finally, the southern giraffe (*G. giraffa*) included 11 individuals from its two subspecies: the Angolan (*G. g. angolensis*) and the South African giraffe (*G. g. giraffa*). For quality control of short-reads we used FastQC version 0.11.7 (www.bioinformatics.babraham.ac.uk/projects/fastqc/) and Trimmomatic version 0.38 [64] with the options ‘ILLUMINACLIP:TruSeq3-PE-2.fa:2:30:10’, ‘SLIDINGWINDOW:4:20’, and ‘MINLEN:40’. To map the reads of each low-coverage genome individual onto the Kordofan giraffe genome assembly, we used BWA-MEM version 0.7.17-r1188 [65]. We sorted the resulting BAM files using Samtools version 1.9 [66] and marked duplicates with the MarkDuplicates tool from Picard version 2.18.21 (http://broadinstitute.github.io/picard/). The mapped BAM files from the 48 giraffe individuals [18] have a mean coverage of 19.5X (7-31X) and a mean insert size of 310 bp (247-515 bp). We removed data from two individuals from the initial data set due to excess deletions or other issues: ENP11 (*G. g. giraffa*) and MF24 (*G. c. camelopardalis*); resulting in a data set of 48 individuals.

To identify TE insertions across the giraffe population, we used MELT version 2.2.0 [20]. As the reference genome is nested inside the analyzed population, we used both the MELT-Split and MELT-Deletion pipelines. MELT-Split screens for TEs absent from the reference assembly but present in the analyzed individual (REF- insertions). MELT-Deletion, in contrast, identifies TEs that are present in the reference genome but not in the individual (REF+ insertions). We used the two species-specific L1-1_Gir and RTE-1_Gir consensus sequences together with the RepeatMasker annotation for each TE consensus sequence to create individual mobile element insertion (MEI) files for MELT-Split. As recommended by the MELT authors, we used a substitution rate of 3 out of 100 nucleotides for LINEs. To speed up the analysis, we ran several instances of MELT in parallel using GNU Parallel [67]. We inverted MELT-Deletion calls so that 0 means insertion present, while 1 means insertion absent, as in [27], as the starting point are annotated TEs in the reference genome.

MELT implements several strict internal filters and removes TE calls in and near N and tandem repeated regions [20]. We used three additional criteria to filter the MELT TE calls to reduce the number of false positives and spurious detections: We removed TE calls that: (1) did not pass MELT internal filters, (2) had less than five read pairs on each side supporting the insertion, and (3) were less than 100 bp in length. These filters are implemented in the analysis RMarkdown script in the Gitlab repository (see data and materials). We inferred the amount of heterozygous TE insertions directly from the filtered MELT insertions, which classifies insertions as homozygous (1/1) or heterozygous (0/1) based on the read coverage of each locus. To test for significant differences in heterozygosity among the four giraffe species and subspecies, we ran a phylogeny-informed ANOVA using the function phylANOVA from the phytools package [68]. All data wrangling and plotting was done with functions from the tidyverse set of R packages [69].

### Phylogenetic analysis of TE insertions

We mapped TE insertions to the phylogenetic tree from [18]. We used ggtree [70] to import the phylogeny, and the vcfR package [71] to import the VCF data sets. A combination of phytools [68], ggtree, and custom-written functions were used to map each TE insertion to its specific branch in the phylogeny for plotting and for extracting the insertion age based on the branch lengths of the tree. In addition, we used the TE insertions to create a species network and phylogeny. We coded insertions for presence (1) and absence (0) at each locus for each of the individuals. We coded heterozygous insertions as presence (1). The okapi was included as an artificial outgroup coded as 0 for all loci. Using the ape package [72], we transformed the data set into a matrix in Nexus format. We used SplitsTree4 version 4.16.2 [73] to calculate a NeighborNet and a Neighbor-Joining phylogeny using default parameters. PAUP version 4.0a build 169 (available at https://paup.phylosolutions.com/) [32] was used to reconstruct a parsimony tree using the Irrev.up character type, which is suitable for TEs which are irreversibly inserted and rarely removed. The heuristic tree search was run with random addition of sequences and 100 repetitions using Tree Bisection and Reconnection. One thousand bootstrap replicates were used to calculate support values. Conflicting TE insertions were visualized using an UpSet plot [74] as implemented in UpSetR [75].

### Comparison between TE- and SNP-based population inferences

Population-genetic analyses were performed in R-4.1.0 [76] using wrapper functions of the R package SambaR [77]. The data was imported into R and stored in a genlight object provided by the R package adegenet [78, 79]. Nei’s genetic distances (D), between all individual pairs and all population pairs, were calculated with the function ‘stamppNeisD’ of the R package StAMPP [80]. Pairwise population Weir & Cockerham 1984 Fst estimates were calculated with the function ‘stamppFst’ of the R package StAMPP. Pairwise population Fst-values according to Wright 1943 were calculated with the function ‘runWrightFst’ of the R package SambaR [77]. Principal component analyses (PCA) was performed using the function ‘snpgdsPCA’ of the R package SNPRelate [81]. Principal coordinate analyses (PCoA) was performed using the function ‘pcoa’ of the R package ape [72], based on a matrix of Nei’s genetic distances between individuals. Neighbourhood joining clustering was performed using the function ‘NJ’ of the R package phangorn [82], using as input a Hamming’s genetic distance matrix between individuals, calculated with the function ‘bitwise.dist’ of the R package poppr [83]. Individual heterozygosity estimates were obtained by estimating the proportion of heterozygous genotypes per individual, after excluding missing data points, using the formula: n1/(n0 + n1 + n2), in which n0, n1 and n2 represent the number of genotypes with zero, one and two minor allele copies respectively. Two estimates were generated:

- TE-He_all_: an estimate over all markers, also known as multi locus heterozygosity (MLH).
- TE-He_seg_: an estimate over segregating markers (i.e., not including markers which are monomorphic in the population to which the individual has been assigned).

All analyses were performed on four different datasets: the LINE dataset (8764 markers), the RTE dataset (620 markers), the combined TE dataset (9384 markers), and a SNP-dataset (48,046 markers). This 48 K SNP-dataset was obtained by thinning a 730 K SNP dataset generated by an earlier study [18]. The thinning was performed by selecting at maximum 1 SNP every 40 kb, using vcftools [84]. The TE He-estimates were compared to genome wide heterozygosity estimates generated by an earlier study [18].

## Supplementary Information

**Additional file 1.** Supplementary text, Supplementary Tables 1-9, Supplementary Figures 1-10.

**Additional file 2.** Fasta consensus sequences of L1-1_Gir, RTE-1_Gir and associated SINEs.

**Additional file 3.** Results of the clustering analysis of SINE activity in the Kordofan giraffe genome.

## Data Availability

The datasets generated and/or analysed during the current study are available in the Dryad repository at 10.5061/dryad.ksn02v74f. This includes: a) the TE annotation of the Kordofan giraffe genome assembly, b) the results of the SINE clustering analysis, c) the results of the TE insertion analysis in the giraffe individuals, and d) genotype files containing with 9384 TE markers and 48046 SNP markers. The whole-genome shotgun sequencing read data are deposited at SRA under the bioproject PRJNA635165, and additional information is documented in [18]. The RMarkdown document for the analyses and other supplementary scripts are available at https://gitlab.com/mpetersen/giraffe-tes. The R commands for the analyses of the genotype files are available from the Dryad repository.
