# Supplementary material for: Population analysis of retrotransposons in giraffe genomes supports RTE decline and widespread LINE1 activity in Giraffidae
Source: Mob DNA. 2021 Nov 26;12:27. doi: 10.1186/s13100-021-00254-y (PMC8620236; doi:10.1186/s13100-021-00254-y)
Supplement: Supplementary file 1 — Additional file 1. Supplementary text, Supplementary Tables 1-9, Supplementary Figures 1-10. [file 13100_2021_254_MOESM1_ESM.docx]

Population analysis of retrotransposons in giraffe genomes supports RTE decline and widespread LINE1 activity in Giraffidae

Supplementary Material

Malte Petersen, Sven Winter, Raphael Coimbra, Menno de Jong, Vladimir V. Kapitonov, Maria A. Nilsson

Supplementary Text

Supplementary Tables 1-9

Supplementary Figures 1-10

# Supplementary text

## Introduction

The main fraction of TEs in mammalian genomes is formed by non-LTR retrotransposons composed of autonomous and non-autonomous elements, known as LINEs and SINEs (Long and Short Interspersed Nuclear Elements), respectively. LINEs, based on their structure and phylogeny of the reverse transcriptase domain universally encoded by them, belong to 28 different clades [1,2]. Mammals were colonized by LINEs that belong to only 3 different clades: L1, L2, and RTE; all these clades have originated above 600 million years ago (MYA). The L2/LINE2 elements have been extremely prolific in ancient mammals >100 MYA [3]; however, they appear to be extinct now in all therians and remain active in monotremes only [4]. In most mammals, the active LINE is the so-called LINE1/L1 element that belongs to the L1 clade. The human genome contains about 500,000 copies of L1 [5], similar to most mammalian genomes [6], excluding monotremes, where L1s are not present [4,7]. Among these 500,000 L1 copies, only 80-100 copies are active retrotransposons [8]. The number of active L1s in the genome can vary between mammalian species [6]. The human genome harbors about one million primate-specific SINEs (Alus), but only about 1800 copies can propagate [9]. The number of active LINEs and SINE copies in the genome of mammals other than humans is generally unexplored.

## TE content of the giraffe genome

We derived the two autonomous giraffe-specific consensus sequences RTE-1_Gir (3,872 bp) and L1-1_Gir (7,997 bp) from the Kordofan giraffe assembly by using either a full-length cattle (*Bos taurus*) LINE1 or searching for coding ORF2s (Supplementary Figure 1, Supplementary Data 1) with two different approaches (see Methods). The cattle RTE (named BovB), and giraffe RTE consensus sequence (RTE-1_Gir) are overall 91% identical to each other. The major 3’ terminal part (pos. 544–3847) is 95% identical to the corresponding part of RTE-1_Gir (pos. 568–3872). However, the 5’ UTR (pos. 1-560) of cattle and giraffe RTE consensus sequences share only 67% identity. The same analysis of the giraffe LINE1 consensus sequence (L1-1_Gir) shows that the 7,997 bp L1-1_Gir sequence is 86% identical to the recently active 8,390 bp L1-BT cattle retrotransposon, which is a recent LINE1 insertion and not a consensus sequence [10].

## References

1. Malik HS, Burke WD, Eickbush TH. The age and evolution of non-LTR retrotransposable elements. Mol Biol Evol. 1999;16(6):793-805. doi: 10.1093/oxfordjournals.molbev.a026164.

2. Kapitonov VV, Tempel S, Jurka J. Simple and fast classification of non-LTR retrotransposons based on phylogeny of their RT domain protein sequences. Gene. 2009;448:207-13. doi: 10.1016/j.gene.2009.07.019.

3. Smit AF. Interspersed repeats and other mementos of transposable elements in mammalian genomes. Curr Opin Genet Dev. 1999;9:657-63.

4. Warren WC, Hillier LW, Marshall Graves JA, Birney E, Ponting CP, Grützner F, et al Genome analysis of the platypus reveals unique signatures of evolution. Nature. 2008;453:175-83. doi: 10.1038/nature06936.

5. Lander ES, Linton LM, Birren B, Nusbaum C, Zody MC, Baldwin J, et al. Initial sequencing and analysis of the human genome. Nature. 2001;409:860–921.

6. Ivancevic AM, Kortschak RD, Bertozzi T, Adelson DL. LINEs between Species: Evolutionary Dynamics of LINE-1 Retrotransposons across the Eukaryotic Tree of Life. Genome Biol Evol. 2016;8:3301–22.

7. Ivancevic AM, Kortschak RD, Bertozzi T, Adelson DL. Horizontal transfer of BovB and L1 retrotransposons in eukaryotes. Genome Biol. 2018;19:85.

8. Brouha B, Schustak J, Badge RM, Lutz-Prigge S, Farley AH, Moran JV, et al. Hot L1s account for the bulk of retrotransposition in the human population. Proc Natl Acad Sci U S A. 2003;100:5280–5.

9. Bennett EA, Keller H, Mills RE, Schmidt S, Moran JV, Weichenrieder O, et al. Active Alu retrotransposons in the human genome. Genome Res. 2008;18:1875-83. doi: 10.1101/gr.081737.108.

10. Girardot M, Guibert S, Laforet MP, Gallard Y, Larroque H, Oulmouden A. The insertion of a full-length *Bos taurus* LINE element is responsible for a transcriptional deregulation of the Normande Agouti gene. Pigment Cell Res. 2006;19:346-55. doi: 10.1111/j.1600-0749.2006.00312.x.

# Supplementary tables

**Supplementary Table 1.** **TEs in the Kordofan giraffe genome.**

| TE | % of genome | Copy number |
| --- | --- | --- |
| SINE | 3.73 | 642,964 |
| LINE | 30.47 | 2,605,207 |
| LTR elements | 5.08 | 408,695 |
| DNA elements | 2.78 | 397,077 |
| Unclassified | 1.77 | 73,573 |
| Small RNA | 1.73 | 261,602 |
| Satellites | 0.07 | 3,256 |
| Simple repeats | 0.72 | 407,527 |
| Low complexity | 0.14 | 70,772 |
| Total | 44.57 |  |

**Supplementary Table 2. Population sampling of giraffe used in the study.**

| Label | Taxonomy (Fennessy et al., 2016) | Platform | Design | Total reads (M) | Insert Size | Raw coverage |
| --- | --- | --- | --- | --- | --- | --- |
| WA720 | *G. camelopardalis peralta* | Illumina | 350 bp (2 x 150 bp) | 399,1 | 278 bp | 22 |
| WA733 | *G. camelopardalis peralta* | Illumina | 350 bp (2 x 150 bp) | 299,1 | 312 bp | 16 |
| WA746 | *G. camelopardalis peralta* | Illumina | 300 bp (2 x 150 bp) | 257,4 | 285 bp | 14 |
| WA806 | *G. camelopardalis peralta* | Illumina | 350 bp (2 x 150 bp) | 378,8 | 282 bp | 21 |
| WA808 | *G. camelopardalis peralta* | Illumina | 350 bp (2 x 150 bp) | 335,1 | 305 bp | 18 |
| GNP01 | *G. camelopardalis antiquorum* | Illumina | 350 bp (2 x 150 bp) | 328 | 305 bp | 18 |
| GNP04 | *G. camelopardalis antiquorum* | Illumina | 350 bp (2 x 150 bp) | 364,7 | 317 bp | 20 |
| GNP05 | *G. camelopardalis antiquorum* | Illumina | 350 bp (2 x 150 bp) | 404,3 | 292 bp | 22 |
| SNR2 | *G. camelopardalis antiquorum* | Illumina | 350 bp (2 x 150 bp) | 340,1 | 304 bp | 18 |
| ZNP01 | *G. camelopardalis antiquorum* | Illumina | ??? bp (2 x 125 bp) | 476,6 | 474 bp | 22 |
| ETH1 | *G. camelopardalis camelopardalis* | Illumina | 300 bp (2 x 150 bp) | 371,8 | 289 bp | 19 |
| ETH2 | *G. camelopardalis camelopardalis* | Illumina | 350 bp (2 x 150 bp) | 334,2 | 301 bp | 18 |
| ETH3 | *G. camelopardalis camelopardalis* | Illumina | 350 bp (2 x 150 bp) | 337,3 | 300 bp | 19 |
| MF06 | *G. camelopardalis camelopardalis* | Illumina | 300 bp (2 x 150 bp) | 309,9 | 283 bp | 17 |
| MF22 | *G. camelopardalis camelopardalis* | Illumina | 350 bp (2 x 150 bp) | 522,5 | 320 bp | 29 |
| RET1 | *G. reticulata* | Illumina | 350 bp (2 x 150 bp) | 398,7 | 311 bp | 22 |
| RET3 | *G. reticulata* | Illumina | 300 bp (2 x 150 bp) | 287 | 286 bp | 15 |
| RET4 | *G. reticulata* | Illumina | 350 bp (2 x 150 bp) | 455,5 | 325 bp | 26 |
| RET5 | *G. reticulata* | Illumina | 350 bp (2 x 150 bp) | 411,4 | 318 bp | 23 |
| RET6 | *G. reticulata* | Illumina | 300 bp (2 x 150 bp) | 390,4 | 289 bp | 20 |
| RETRot1 | *G. reticulata* | Illumina | 350 bp (2 x 150 bp) | 437,5 | 300 bp | 24 |
| RETRot2 | *G. reticulata* | Illumina | 350 bp (2 x 150 bp) | 396,8 | 320 bp | 22 |
| ISC01 | *G. reticulata* | Illumina | 350 bp (2 x 150 bp) | 306,3 | 247 bp | 7 |
| ISC04 | *G. reticulata* | Illumina | 350 bp (2 x 150 bp) | 311,6 | 281 bp | 17 |
| ISC08 | *G. reticulata* | Illumina | 300 bp (2 x 150 bp) | 391,7 | 288 bp | 20 |
| LVNP8-04 | *G. tippelskirchi thornicrofti* | Illumina | 300 bp (2 x 150 bp) | 234,3 | 278 bp | 11 |
| LVNP8-08 | *G. tippelskirchi thornicrofti* | Illumina | 350 bp (2 x 150 bp) | 312,1 | 290 bp | 17 |
| LVNP8-09 | *G. tippelskirchi thornicrofti* | Illumina | 350 bp (2 x 150 bp) | 381,3 | 296 bp | 18 |
| LVNP8-10 | *G. tippelskirchi thornicrofti* | Illumina | 350 bp (2 x 150 bp) | 322 | 287 bp | 14 |
| LVNP8-12 | *G. tippelskirchi thornicrofti* | Illumina | 350 bp (2 x 150 bp) | 305,1 | 293 bp | 16 |
| LVNP8-14 | *G. tippelskirchi thornicrofti* | Illumina | 350 bp (2 x 150 bp) | 368 | 320 bp | 21 |
| MA1 | *G. tippelskirchi* | Illumina | 550 bp (2 x 150 bp) | 415,7 | 515 bp | 22 |
| SGR01 | *G. tippelskirchi* | Illumina | 350 bp (2 x 150 bp) | 315,7 | 321 bp | 18 |
| SGR05 | *G. tippelskirchi* | Illumina | 350 bp (2 x 150 bp) | 504,7 | 315 bp | 27 |
| SGR07 | *G. tippelskirchi* | Illumina | 350 bp (2 x 150 bp) | 378 | 280 bp | 21 |
| SGR13 | *G. tippelskirchi* | Illumina | 350 bp (2 x 150 bp) | 410,3 | 317 bp | 23 |
| SGR14 | *G. tippelskirchi* | Illumina | 350 bp (2 x 150 bp) | 542,2 | 329 bp | 31 |
| BNP02 | *G. giraffa giraffa* | Illumina | 350 bp (2 x 150 bp) | 295,6 | 289 bp | 16 |
| KKR01 | *G. giraffa giraffa* | Illumina | 350 bp (2 x 150 bp) | 335,6 | 310 bp | 19 |
| KKR08 | *G. giraffa giraffa* | Illumina | 300 bp (2 x 150 bp) | 316 | 304 bp | 17 |
| MTNP09 | *G. giraffa giraffa* | Illumina | 350 bp (2 x 150 bp) | 326,5 | 279 bp | 18 |
| SUN3 | *G. giraffa giraffa* | Illumina | 350 bp (2 x 150 bp) | 320,5 | 313 bp | 18 |
| V23 | *G. giraffa giraffa* | Illumina | 350 bp (2 x 150 bp) | 348,4 | 320 bp | 19 |
| ENP16 | *G. giraffa angolensis* | Illumina | 350 bp (2 x 150 bp) | 372 | 331 bp | 21 |
| ENP19 | *G. giraffa angolensis* | Illumina | 350 bp (2 x 150 bp) | 418,8 | 319 bp | 24 |
| ENP20 | *G. giraffa angolensis* | Illumina | 350 bp (2 x 150 bp) | 339,9 | 329 bp | 19 |
| HNB102 | *G. giraffa angolensis* | Illumina | 350 bp (2 x 150 bp) | 362,7 | 302 bp | 20 |
| HNB110 | *G. giraffa angolensis* | Illumina | 350 bp (2 x 150 bp) | 340 | 322 bp | 19 |

**Supplementary Table 3**. **Average heterozygosity ratio per species/subspecies.** Heterozygosity for each TE, as well as overall heterozygosity of all insertions (combined).

| Species | L1-1_Gir | RTE-1_Gir | All |
| --- | --- | --- | --- |
| Northern giraffe | 30.58 | 22.60 | 26.53 |
| -Kordofan | 32.10 | 22.80 | 27.44 |
| -Nubian | 31.05 | 22.23 | 26.60 |
| -West African | 28.52 | 22.79 | 25.55 |
| Reticulated giraffe | 42.94 | 29.53 | 36.06 |
| Southern giraffe | 30.02 | 20.11 | 25.52 |
| -Angolan | 32.47 | 20.93 | 26.70 |
| -South African | 29.76 | 19.44 | 24.54 |
| Masai giraffe | 31.94 | 20.95 | 25.93 |
| -Masai sensu. str | 41.29 | 26.32 | 33.59 |
| -Luangwa | 21.30 | 15.33 | 18.26 |

**Supplementary Table 4**. **Number of full-length L1-1_Gir and RTE-1_Gir copies***.

| Species | heterozygous  L1-1_Gir | homozygous  L1-1_Gir | All  L1-1_Gir | All  RTE-1_Gir |
| --- | --- | --- | --- | --- |
| Northern giraffe | 855 | 497 | 1352 | 3 |
| -Kordofan | 309 | 162 | 471 | / |
| -Nubian | 286 | 160 | 446 | / |
| -West African | 260 | 175 | 435 | / |
| Reticulated giraffe | 1082 | 336 | 1418 | 10 |
| Southern giraffe | 1003 | 815 | 1818 | 21 |
| -Angolan | 489 | 383 | 872 | / |
| -South African | 514 | 432 | 946 | / |
| Masai giraffe | 1024 | 826 | 1850 | 25 |
| -Masai sensu. str | 702 | 361 | 1063 | / |
| -Luangwa | 322 | 465 | 787 | / |
| Total | 3964 | 2474 | 6438 | 59 |

*L1-1_Gir SVLEN > 7980 bp/RTE-1_Gir SVLEN > 3850 bp

**Supplementary Table 5.** **Copy number of full-length RTE-1_Gir ORF in three giraffe assemblies.** MA1 and OR1865 stem from the same individual but have been assembled with different techniques.

|  | Kordofan  (GCA_018282235.1) | Masai MA1 (GCA_001651235.1) | Masai OR1865 (GCA_006408565.1) |
| --- | --- | --- | --- |
| Full-length ORF | 3049 | 2547 | 1126 |
| Intact RTE ORF | 9 | 5 | 0 |

**Supplementary Table 6.** **Heterozygosity for selected nodes in the giraffe phylogeny (see figure 3 for node numbers).**

| node | heterozygous | homozygous | Total | % het |
| --- | --- | --- | --- | --- |
| 50 | 1799 | 2706 | 4505 | 39.9 |
| 51 | 1217 | 368 | 1585 | 76.7 |
| 52 | 255 | 130 | 385 | 66.2 |
| 66 | 290 | 66 | 356 | 81.5 |
| 75 | 362 | 450 | 812 | 44.6 |
| 76 | 459 | 475 | 934 | 49.1 |
| 86 | 422 | 456 | 878 | 48.1 |

het: heterozygosity

**Supplementary Table 7. Heterozygosity of full-length* L1-1_Gir and RTE-1_Gir for nodes in the giraffe phylogeny (see figure 3 for node numbers)**.

| node | L1-1_Gir  0/1 | L1-1_Gir  0/0 | L1-1_Gir  Total | L1-1_Gir  % het |
| --- | --- | --- | --- | --- |
| 50 | 45 | 43 | 88 | 51.1 |
| 51 | 92 | 21 | 113 | 81.4 |
| 52 | 23 | 12 | 35 | 65.7 |
| 66 | 45 | 3 | 48 | 93.7 |
| 75 | 21 | 25 | 46 | 45.7 |
| 76 | 85 | 45 | 130 | 65.4 |
| 86 | 72 | 36 | 108 | 66.7 |

*L1-1_Gir SVLEN > 7980 bp/RTE-1_Gir SVLEN > 3850 bp

**Supplementary Table 8.** **Amount of full-length* LINE1 insertions for nodes in the giraffe phylogeny (see figure 3 for node numbers).**

| node | All copies at node | FL L1 | % FL |
| --- | --- | --- | --- |
| 50 | 4505 | 88 | 1.9 |
| 51 | 1585 | 113 | 7.1 |
| 52 | 385 | 35 | 9.1 |
| 66 | 356 | 48 | 13.5 |
| 75 | 812 | 46 | 5.6 |
| 76 | 934 | 130 | 13.9 |
| 86 | 878 | 108 | 12.3 |

*SVLEN > 7980 nt

**Supplementary Table 9. Population differentiation estimates for all pairwise comparisons of giraffe subspecies.** Above diagonal: Nei’s genetic distance (Nei, 1972). Below diagonal: Fst-values according to Weir and Cockerham (1984). Estimates are inferred from a 9.4K TE dataset (LINE1 and RTE markers combined).

|  | Luangwa | Masai  Sensu Str | South  African | Angolan | Nubian | Kordofan | West  African | Reticulated |
| --- | --- | --- | --- | --- | --- | --- | --- | --- |
| Luangwa | 0 | 0.065 | 0.203 | 0.22 | 0.233 | 0.234 | 0.238 | 0.213 |
| Masai Sensu Str | 0.268 | 0 | 0.177 | 0.192 | 0.215 | 0.215 | 0.22 | 0.193 |
| South African | 0.577 | 0.492 | 0 | 0.029 | 0.228 | 0.23 | 0.236 | 0.21 |
| Angolan | 0.613 | 0.524 | 0.09 | 0 | 0.249 | 0.249 | 0.255 | 0.23 |
| Nubian | 0.634 | 0.558 | 0.591 | 0.628 | 0 | 0.028 | 0.043 | 0.059 |
| Kordofan | 0.636 | 0.56 | 0.594 | 0.63 | 0.109 | 0 | 0.037 | 0.061 |
| West African | 0.652 | 0.576 | 0.61 | 0.647 | 0.204 | 0.178 | 0 | 0.069 |
| Reticulated | 0.542 | 0.483 | 0.516 | 0.542 | 0.217 | 0.226 | 0.26 | 0 |

# Supplementary figures

**Supplementary Figure 1. Schematic structure of the autonomous L1-1_Gir, RTE-1_Gir and their non-autonomous counterparts (SINEs).** The ORF2 of 7,997-bp L1-1_Gir and single open reading frame of 3,871-bp RTE-1_Gir coding for the DNA endonuclease and reverse transcriptase domains are marked in pink and green, respectively. The tRNA(Glu)-derived region, containing the internal pol III promoter is in red. Similar DNA regions present in different retrotransposons are marked by the same colors. Microsatellite 3’-tails are shown as (CORE)_n_, where CORE is a microsatellite unit and n is the average number of units in the corresponding consensus sequence. In all RTE-dependent SINEs, their 80-95 bp long 3’-terminal regions are similar to the 3’ terminal region of RTE-1_Gir (positions 3793-3861). Two internal deletions at positions 164-1580 or 164-2661 in the L1-1_Gir 5’-noncoding region formed two minor subfamilies, 6579-bp L1-1A_Gir and 5494-bp L1-1B_Gir.

**Supplementary Figure 2.** **Groups or clusters of identical Bov-tA and Bov-A2 retrotransposons identified in the Kordofan giraffe (A, B) and cow (C, D) genomes.** Each plot shows the number of clusters and pseudoclusters composed of a specific number of identical full-length copies of the retrotransposons identified in the genome. Pseudoclusters, clusters composed of the retrotransposon copies that are internal parts of segmental duplications, are shown in the right bin halves (green). Clusters composed of the TEs copies residing outside segmental duplications are shown in left bin halves (black). The number of clusters and pseudoclusters are shown above the corresponding bars.

**Supplementary figure 3.** **Length distribution of LINE1 copies in giraffe genomes.**

The length in basepairs (bp) of the detected LINE1 copies across the four giraffe species. The majority of copies are short, but there is a large number of copies that are close to the consensus sequence length of L1-1_Gir (7,997 bp).

**Supplementary Figure 4.**  **Schematic evolution of “intact-like” RTEs identified in the giraffe genome assemblies.** RTEs coding for the intact ORFs are marked by the plain circles: nine in the Kordofan (blue, K1 to K9) and five in the Masai MA1 giraffe genome (green, M1 to M5). Each intact element and its orthologous copies are shown in the same row. Circles with black borders mark RTE elements that are not fully represented in the assemblies or are cut off at contig boundaries (“N-gaps”). The crossed circles mark elements with the defective ORFs. Black squares mark empty targets, e.g., absent RTE elements. The question mark indicates that the M3 flanking sequences are not present in the okapi genome assembly. The “N” letter indicates that a ~5 kbp region containing the orthologous flanks of M1 is absent in the Kordofan giraffe assembly. MA1 and OR1865 are two different assemblies of the same genomic data from a single individual of the Masai giraffe. One phylogeny-conflicting insertion of RTE (K1) is marked by grey double lines.

**Supplementary figure S5.** **Phylogenetic reconstruction of giraffe species from transposable element (TE) insertions.** Phylogenetic trees of the four giraffe species reconstructed on a data set of 9,382 TE loci. A) Parsimony phylogeny reconstructed with PAUP. B) NeighborNetwork reconstructed with SplitsTree. C) Neighbor-joining phylogeny reconstructed with SplitsTree. The phylogenies are rooted on Okapi (WOAK), and the common names of the four giraffe species are indicated.

**Supplementary Figure S6.** LINE1 insertions mapped to the branches of the giraffe phylogeny. Numbers on the branches indicate the number of insertions traced to the most recent common ancestor (MRCA) of the individuals that have the specific insertion. Branch widths and colors indicate the number of insertions scaled to the branch lengths.

**Supplementary Figure S7.** RTE insertions mapped to the branches of the giraffe phylogeny. Numbers on the branches indicate the number of insertions traced to the most recent common ancestor (MRCA) of the individuals that have the specific insertion. Branch widths and colors indicate the number of insertions scaled to the branch lengths.

**Supplementary figure 8.** Biplots showing the first three axes of principal coordinate analyses (PCoA) for A) a 48K SNP dataset; B) a 9.8K TE dataset (LINE1 and RTE combined); C) a 8.7K LINE1 dataset; and D) a 0.6K RTE dataset.

**Supplementary figure 9.** Scatterplot showing sample specific heterozygosity estimates inferred from a 9.4K TE dataset (y-axis) against genome-wide heterozygosity (He) estimates (x-axis) reported by Coimbra et al. (2021). Excluded from the TE-He calculation are monomorphic markers (i.e., markers which are not segregating in the population to which the individual belongs).

**Supplementary figure 10.** Idem as main figure 4C. Dots have been replaced with sample names. He: heterozygosity.
