# Supplementary material for: Population analysis of retrotransposons in giraffe genomes supports RTE decline and widespread LINE1 activity in Giraffidae
Source: Mob DNA. 2021 Nov 26;12:27. doi: 10.1186/s13100-021-00254-y (PMC8620236; doi:10.1186/s13100-021-00254-y)
Supplement: Supplementary file 2 — Additional file 2. Fasta consensus sequences of L1-1_Gir, RTE-1_Gir and associated SINEs. [file 13100_2021_254_MOESM2_ESM.docx]

Supplemental data set 1

Consensus sequences of RTE-1_Gir and L1-1_Gir as well as giraffe-specific versions of SINEs

These are also available as Fasta files in the Dryad repository (data package 1) for this study.

**>RTE-1_Gir**

CTGCGACGGCTCGGCGCAGAAGCGAGGCGGCTGCGACGGCTCGGCGCAGAAGCGTGGCCGAGAGGAGCTA

GGCCGGGCCGGGGGCCGGGGGCGGCAACCGAGAGGGGCTAGGCCGGGCCGGGGGCCGGCGGCGGCCGACA

GGGGCTAGGCCGGGCCGGGGGCCGGGGGCCGGGGGCCGGGGGCGGCGGCCGAGAGGGGCTAGGCCGGGCC

GGGGGCCGGGGGCGGCGGCCGACAGGGCCTAGGCCGGGCCGGGGGCCGGGAGCCGGGGGCGGCGGCCGAG

AGGGGCTAGGCCGGGCCGGGGGCCGGGGGCCGGGGGCGGCGGCCGACAGGGGCTAGGCCGGGCCGGGGGC

CGGGAGCTGGGGGCGGCGGCCGAGAGGGGCTAGGCCGGGCCGGGGGCCGGGGGCCGGGGGCGGCGGCCGA

CAGGGGCTAGGCCGGGCCGGGGGCCGGGGGCAGCGGCTGACAGGGCCTAGGCCGGGCCGGGGGCCGGGGG

CCGGGGGCCGGGGGCGGCAGCCGAGAGGGGCTAGGCCAGGCCGGGGGCTGGGGCCGGCGGCGGCGGTGGC

GGCGGCAAGAGGAATTACCCAAGGCCCAAGGTAAGAGAAACCCAAGTAAGACGGTAAGTGTTGCGAGAGG

GCATCAGAGGGCAGACACACTAAAACTAAAACTACAGAAAACTAGCTAATCTGATCACACGGACCACAGC

CTTGTCTATCTCAATGAAACTAAGCCATGCCGTGTGGGGCCACCCAAGACGGATGGGTCATGGTGGAGAG

GTCTGACAGATTGTGGTCCACTGGAGAAGGGCATGGCAAACCACTTCAGTATTCTTGCCTTGAGAACCCC

ATGAACAGTATGAAAAGGCAAAAGGATAGGACACTGAAAGATGAACTCCCCAGGTCAGTAGGTGCCCAAT

ATGCTAATGGAGATCAGTGGAGAAATAACTCCAGAAAGAATGATGGGATGGAGCCAAAGCAAAAACGATA

CCCAACTGTGGATGTGACTGGTGATAGACGCAAAGTCCGATGCTGTAAAGAGCAATATTGCATAGGAACC

TGGAATGTTAGGTCCATGAATCAAGGCAAACTGGAAGTGGTCAAACAGGAGATGGCAAGAGTGAACGTTG

ACATTCTAGGAATCAGCGAACTAAGATGGACTGGAATGGGTGAATTTAACTCAGATGACCATTATATCTA

CTACTGTGGGCAAGAATCCCTTAGAAGAAATGGAGTAGCCATCATGGTCAACAAAAGAGTCCGAAATGCA

GTACTTGGATGCAATCTCAAAAACGACAGAATGATCTCTGTTCGTTTCCAAGGCAAACCATTCAATATCA

CGGTAATCCAAGTCTATGCCCCGACCAGTAACGCCGAAGAAGCTGAAGTTGAACGGTTCTATGAAGACCT

ACAAGACCTTCTAGAACTAACACCCAAAAAAGATGTCCTTTTCATTATAGGGGACTGGAATGCAAAAGTA

GGAAGTCAAGAAACACCTGGCGTAACAGGCAAGTTTGGCCTTGGAGTACAGAATGAAGCAGGGCAAAGGC

TAATAGAGTTTTGCCACGAGAACGCACTGGTAATAGCAAACACCCTATTCCAACAACACAAGAGAAGACT

CTACACGTGGACATCACCAGATGGTCAACACCGAAATCAGATTGATTATATTCTTTGCAGCCAAAGATGG

AGAAGCTCTATACAGTCAGCAAAAACAAGACCAGGAGCAGACTGTGGCTCAGATCATGAACTGCTTATTG

CCAAATTCAGACTTAAATTGAAGAAAGTAGGGAAAACCACTAGACCATTCAGGTATGACCTAGATCAAAT

CCCTTATGAGTATACAGTGGAAGTGACAAATAGATTTAAGGGACTAGACCTGATAGACAGAGTGCCTGAT

GAACTATGGACAGAGGTTCGTGACATTGTACAGGAGACAGGGATCAAGAAAATCCCCATGGAAAAGAAAT

GCAAAAAAGCAAAATGGCTGTCTGAGGAGGCCTTACAGATAGCTGGGAAGAGAAGAGAAGCGAAAAGCAA

AGGAGAAAAGGAAAGATATTCCCATCTGAATGCAGAGTTCCAAAGAATAGCAAGGAGAGATAAGAAAGCC

TTCCTCAGCGATCAGTGCAAACAAATAGAGGAAAACAACAGATTGGGAAAGACTAGAGACCTCTTCAAGA

AAATTAGAGATACCAAAGGAACATTTCATGCAAAGATAGGCTCGATAAAGGACAGAAATGGTATGGACCT

AACAGAAGCAGAAGATATTAAGAAGAGGTGGCAAGAATACACAGAAGAACTGTACAAAAAAGATCTTCAC

GACCCAGATAATCACGATGGTGTGATCACTCACCTAGAGCCAGACATCCTGGAATGTGAAGTCAAGTGGG

CCTTAGAAAGCATCACTACGAACAAAGCTAGTGGAGGTGATGGAATTCCAGTTGAGCTATTTCAAATCCT

CAAAGGTGATGCTGTGAAAGTGCTGCACTCGATATGCCAGCAAATTTGGAAAACTCAGCAGTGGCCACAG

GACTGGAAAAGGTCAGTTTTCGTTCCAATCCCAAAGAAAGGCAATGCCAAAGAATGCTCAAACTACCGCA

CAATTGCACTCATCTCACATGCTAGTAAAGTAATGCTCAAAATTCTCCAAGCCAGGCTTCAGCAATACGT

GAACCGTGAACTTCCAGATGTTCAAGCTGGTTTTAGAAAAGGCAGAGGAACCAGAGATCAAATTGCCAAT

ATCCGCTGGATCATCGAAAAAGCAAGAGAGTTCCAGAAAAACATCTATTTCTGCTTTATTGACTATGCCA

AAGCCTTTGACTGTGTGGATCACAATAAACTGTGGAAAATTCTGAAAGAGATGGGAATACCAGACCATCT

GACCTGTCTCTTGAGAAACCTGTATGCAGGTCAGGAAGCAACAGTTAGAACTGGACATGGAACAACAGAC

TGGTTCCAAATAGGAAAAGGAGTACGTCAAGGCTGTATATTGTCACCCTGCTTATTTAACTTATATGCAG

AGTACATCATGAGAAACGCTGGGCTGGAAGAAACACAAGCTGGAATCAAGATTGCCGGGAGAAATATCAA

TAACCTCAGATATGCGGATGACACCACCCTTATGGCAGAAAGTGAAGAAGAACTAAAGAGCCTCTTGATG

AAGGTGAAGGAGGAGAGTGAAAAAGTTGGCTTAAAGCTCAACGTTCAGAAAACTAAGATCATGGCATCTG

GTCCCATCACTCCATGGGAAATAGATGGGGAAACAGTGGAAACAGTGTCAGACTTTACTTTTTTGGGCTC

CAGAATCACTGCTGATGGTGACTGCAGCCATGAAATTAAAAGACGCTTACTCCTTGGAAGGAAGGTTATG

GCCAACCTAGATAGCATGTTTAAAAGCAGAGACATTACTTTGCCAACAAAAGTCCGTCTAGTCAAGGCTA

TGGTTTTTCCAGTGGTCATGTATGGATGTGAGAGTTGGACGGTGAAGAAAGCTGAGCGCCGCAGAATTGA

TGCTTTTGAACTGTGGTGTTGGAGAAGACTCTTGAGAGTCCCTTGGACTGCAAGGAGATCCAATCAATCC

ATCCTAAAAGAGATCAATCCTGGGTGTTCATTGGAAGGTCTGATGTTGAAGCTGAAACTCCAATACTTTG

GCCACCTCATGCGAAGGGTTGACTCATTGGAAAAGACCCTAATGCTGGGTAGGATTGGGGGCGGGAGGAG

AAGGGGACGACAGAGGATGAGATGGTTGGATGGCATCACCGACTCGATGAACATGGGTTTGGGTAGACTC

CGGGAGTTGGTGATGGACAGAGAGGCCTGGCGTGCTGCAATTCATGGGGTCGCAGAGAGTCGGACACGAC

TGAGCGACTGAACTGAACTGA

**>RTE-1A1_**Gir 5'-truncated; some copies have the (AAC)n 3'-terminus instead of (ACTGA)n

CAGTGCCTGAAGAACTATGGACGGAGGTTCGTAACATTGTACAGGAGGCGGTGATCAAAACCATCCCCAA

GAAAAAGAAATGCAAAAAGGCAAAATGGTTGTCTGAGGAGGCCTTACAAATAGCTGAGAAAAGAAGAGAA

GCGAAAGGCAAAGGAGAAAAGGAAAGATATACCCATCTGAATGCAGAGTTCCAAAGAATAGCAAGGAGAG

ATAAGAAAGCCTTCCTCAGTGATCAATGCAAAGAAATAGAGGAAAACAATAGAATGGGAAAGACTAGAGA

TCTCTTCAAGAAAATTAGAGATACCAAGGGAACATTTCATGCAAAGATGGGCACAATAAAGGACAGAAAT

GGTATGGACCTAACAGAAGCAGAAGATATTAAGAAGAGGTGGCAAGAATACACAGAAGAACTATACAAAA

AAGATCTTCATGACCCAGATAACCACGATGGTGTGATCACTCACCTAGAGCCAGACATCCTGGAATGTGA

AGTCAAGTGGGCCTTAGGAAGCATCACTACGAACAAAGCTAGTGGAGGTGATGGAATTCCAGTTGAGCTA

TTTCAAATCCTAAAAGATGATGCTGTGAAAGTGCTGCACTCAATATGCCAGCAAATTTGGAAAACTCAGC

AGTGGCCACAGGACTGGAAAAGGTCAGTTTTCATTCCAATCCCAAAGAAAGGCAATGCCAAAGAATGTTC

AAACTACCACACAATTGCACTCATCTCACATGCTAGCAAAGTAATGCTCAAAATTCTCCAAGCCAGGCTT

CAACAGTACGTGAACCGAGAACTTCCAGATGTTCAAGCTGGATTTAGAAAAGGCAGAGGAACCAGAGATC

AAATTGCCAACATCCGTTGGATCATAGAAAAAGCAAGAGAGTTCCAGAAAAACATCTACTTCTGCTTTAT

TGACTACGCCAAAGCCTTTGACTGTGTGGATCACAACAAACTGTGGAAAATTCTTAAAGAGATGGGAATA

CCAGACCACCTTACCTGCCTCCTGAGAAATCTGTATGCAGGTCAAGAAGCAACAGTTAGAACTGGACATG

GAACAACAGACTGGTTCCAAATTGGGAAAGGAGTACGTCAAGGCTGTATATTGTCACCCTGCTTATTTAA

CTTATATGCAGAGTACATCATGCGAAATGCTGGGCTGGATGAAGCACAAGCTGGAATCAAGATTGCCGGG

AGAAATATCAATAACCTCAGATATGCAGATGACACCACCCTTATGGCAGAAAGCGAAGAGGAACTAAAGA

GCCTCTTGATGAAAGTGAAAGAGGAGAGTGAAAAAGCTGGCTTAAAACTCAACATTCAAAAAACTAAGAT

CATGGCATCCGGTCCCATCACTTCATGGCAAATAGATGGGGAAACAATGGAAACAGTGACAGACTTTATT

TTCTTGGGCTCCAAAATCACTGCAGATGGTGACTGCAGCCATGAAATTAAAAGACGCTTGCTCCTTGGAA

GAAAAGCTATGACCAACCTAGACAGCATATTAAAAAGCAGAGACATTACTTTGCCAACAAAGGTCCGTCT

AGTCAAAGCTATGGTTTTTCCAGTAGTCATGTATGGATGTGAGAGTTGGACCATAAAGAAAGCTGAGCGC

CGAAGAATTGATGCTTTTGAACTGTGGTGTTGGAGAAGACTCTTGAGAGTCCCTTGGACTGCAAGGAGAT

CAAACCAGTCAATCCTAAAGGAAATCAGTCCTGAATATTCATTGGAAGGACTGATGCTGAAGCTGAAACT

CCAATACTTTGGCCACCTGATGCGAAGAACTGACTCATTGGAAAAGACCCTGATGCTGGGAAAGATTGAA

GGCAGGAGGAGAAGGGGATGACAGAGGATGAGATGGTTGGATGGCATCACCGACTCAATGGACATGAGTT

TGAGCAAGCTCCGGGAGTTGGTGATGGACAGGGAAGCCTGGCGTGCTGCAGTCCATGGGGTCGCAAAGAG

TCGGACACGACTGAGCGACTGAACTGAACTGA

**>L1-1_Gir**

GGCGGGGCTAAGATGGCGGAGGAATAGGACGGGGAGACCACTCTCTCCTCCACAAATTTATCGAAAGAAC

ATCTGAACGCTGACCAAACTTCACAAAACTACTTCTGAAGGCTGGCTGAGGACGTCAGGCTTCCACAAAA

ACAGCGCATTATCTTCAAAAGAGGTAGGAGAAATTATAAAAGATAAAAAGAGAGACAAAAGAGTTAGGGA

CGGAGACCCGTCCCGGGGAGGGAGTCGTAAAAGAGGAGGAATTTCCAAGCACCAGGAAACCCTCTCACCG

GCGGGTGTGGGGGAGGATTTTTTATCTCGGAGGGCAACGTAACTGGGAGGAAAAAATAAATAAAATCCAC

AGATTACGTGCCTAAAGCAACTCCCAGCAAAGAACCCCAGACGCTCGCATCATCCGCCACCAGCAAGTGG

GGGCTGAACGGAGAGGAGCGGGCGGCACTGCCTAGGGTAAGGACCGGGCCCGAGTGCCCCGAGGGCAATC

TGAGGGAGCTAACGTGAGATAGTAACCTAAACTGTGGGACAGCGAGAGAGAGAGAACTATCCCGCGAAAA

GCCCAACTCTAAGGCACTGCCGGCCCGCTCACAGAACAAAGGACTGAGCGATACCAGAGGAGAGCTAGCC

GGCTGCGGACCGCCCCATCCCCCGCCGGACACAGCGGGCAGGCGGGGCCCGCCAGAGCCGGAAAGGGGCA

AACTCGGCTCCAGAGAGGGCACCCCCTACCAAACTGCAAACAGGCTCCCAGTTTCTAAGCAAAGACTGCC

TGAGATCCCGGAGGGTCGACAGCCGCCGGGAGGGTCGCAGCCAGAGACCAGCTCCCCAGAACCGACACAA

AGCACACCGGAGCCGGGCGCGCCCGTAAACCGAGGCGGGGACCAGGGAGGTGATAAGCCGCACCGCCCCA

ACAGGGGAGAGCGCCCTCGCCAAACTCCTGGCCGCCTGGGCAGCTCCGACCGGGAACGGCACAAGCCGCG

GGCGAAACCGAGTCTGCGCCTTTGTGGAGTAACCGGGAGCCTGAACCTGAGCGGCTTAGACCTGGGAAGT

GCACGCAGCTCAGGGCCCGCTCCCCGGCCGAGTAACCTGAAGCCTGAGCAGGGTAGACGGGGAAAGCACA

CACGCCGTGAGCGGGGGCGAACCCAGTGCGGCCGAGACACTGCGAGCTTACCACACACACGCCAGAATTG

CAGTGTTCCTTCCTCCCCACAGCACGATTGAACAAGCGAACCTAAAGAGATCACCTCCACCGCTTTGTTG

CAGGGCGGAAATTAGATACTGAAGAGATCTCCAAACAGAAGCCAAATAAACAGAGGGAAGCGCTTTGGAA

GGGACAGGCCAACAGATTAAGATCCCTGTAGTTAATACCGACTGCATTAAAAAGGACCTATAGATCTTGA

AAAGTATAAGCAGGAATAAGGATCTATCTAAAACTGAGCAGATCCCACACTGCTCACAACAGCTCCAGAG

AAAGTCCGGGATATATATATTTTTTACTATTATTATTTTTTTTAATTTTTTTTTAATTTTTTTTTTTAAT

TAAAAAAATTTTTTTTTCTCTTCTTTCCTTTTTCTTCAAGTCCTCTATTACACCTTTAACTTTCATTTTC

ATAACCCACTATTAACTTGCAAAAAAAAAAAAACCCTATTTTTGAAGCAAACTACATACATATATCTTAT

ACCTTTTGTGTTTTCTTTTTTTTAATGTTTTTTTCCTGTTCTACATTTTTTTACTGTTTCTTTTTTTACT

GTTTTACTGTTTTTTTTCTTTTTACACTGTTTCCTTTTTTCTTTTTTTTTTTTTTTTAGATTGTAAGTTT

TATAAGAGTCTAACCTCTACTCTAGATTTTTAATCTTTGTTTTACATATTTGATATCAACTTTGTACATT

TAAGAACCCAATCTTTAGGACCCATTTTTACTCAGGAGTGTGATCACTGGCCTGATCACTCTCTCCCCCT

ATTGATGCTCTTTCTTCTCCCCCAGGGAACCTCTATCTCCCACCCTCCTCCTTCTCTTCTCAACCCAATT

CTGTGAATCTCTGAGGGTGTTCTGGGTACCGGAGAACACTTCAGGAACAGGATACTGCCCAGATCTCTCT

CTCCCCTCTTAATTCCTCCCTTTCTACTCATGCTCACCTCTGTTACCTTCCTCCCCCCTCTCTATGTATC

TCTGTGAATCACTCTGAGGGTTCCAAACTGTGGAAAGCACAGAGGGAAGTGCTTACTGGCTAGATTGCTC

TCTCCACATTTGAATTCCCCTCTTCCCCTCCTGGTCACCTCAACCCACCCCCCCCACCTCATCTCTCCAT

GTAACTCAGTGAACCTTTCTGGGTATCCCTCACTGTGGAGAATCTTTTCACCATTAACCTAAAAGTTTTA

TTATTGAGGCTGTATGGAAGGAGAAGTCTTGAGGCTACTGCAAGAATAAGACTGAAAGCCAGGGGAAACA

TTAATTAATAAGAGACCATCCAATAGCCTCCATAAATTCACTGAAACCAAGCTCCACCCAAGAGACAATA

AGTTCCAGAGCAAGACATACCACGCAAATTCTCCAGCAACGCAGGAACATAGACCTGAGCATCAATATAC

AGGCTGCCCAGAGGCACACCAAATCCATAGACATCTCAAAAATTAATACTGGTCACTCCATTACACTCTA

GAGAGCAGAAATCCAGCTCCACCCACCAGAACACCGACACAAGCTCCCCCAACCAGGAAACCTTGACAAG

CCACCTGTCCAACCCCAACCGCAGTGAGGAACCTCCACAACAAAGAGGAACTTCATACTACCAGAATACA

GAAGGGCCACGCCTAACACAGCAATCTAAACAAGATGAAAAGGCAGAGAAATACTCAGCAAGTAAAGGAA

AATGAGAAATGCCCACAAAGCCAAACAAAAGAGGAGGAGATAGGGAATCTATCTGAAAAAGAATTTAGAA

TAAGGATAATAAAAATGATCCAAAATCTTGAAAGCAAAATGGAGTTACAGATAAATAGCCTGGAGACAAA

GATTGAGAAGATGCAAGAAATGTTTATCAAGGACTTAGAAGAAATAAAAAGGAATCAATATAGAATGAAT

AATGAAATAAAGGAGATAAAAGACACTCTGGAGGGAACCAACAGTAGAATAACGGAGGCAGAAGATAGGA

TAAGTGAGGTAGAAGATAGAATGATGGAAATAAACGAAGCAGAGAGGAAAAAAGAAAAAAGAATCAAAAG

AAATGAGGAAAACCTCCGGGACCTCTGGGACAATATGAAACGCCCCAACATTCGAATCATAGGAGTCCCA

GAAGAAGAAGATAAAAGGAAAGGCCATGAAAAAATACTCGAGGAGATAATAGTTGAGAATTTCCCTAAAT

TGAGGAAAGAAATAGTCACAGAAGTCCAAGAAACCCAGAGAGTTCCAAACAGGATAAACCCAAGGCGAAA

CACCCCAAGACACATATTAATCAAATTAACAAAGATCAAACACAAAGAACAAATATTAAAAGCAGCAAGG

GAAAAACAACAAATAACACACAAGGGGATCCCCATAAGGATAACAGCTGATCTTTCAATAGAAACCCTTC

AGGCTAGAAGGGAATGGCAGGACATACTTAAAGTAATGAAAGAGAATAATCTACAACCCAGATTACTGTA

CCCAGCAAGGATCTCATTCAGATATGAAGGAGAATTCAAAAGCTTTACAGACAAGCAAGAGCTGAGGGAA

TTCAGCACCACCAAACCAGCTCTCCAGCAAATGCTAAAGGATCTTCTCTAAACAGGAAACACAGAAAGGA

TGTTTCAACACGAACTCAAAACAACAAAGTAAATGGCAACGGGATCATACTTATCAATAATTACCTTAAA

TGTAAATGGGTTGAATGCCCCAACCAAAAGACAAAGGGTTGCTGAATGGATACAAAAACAAGACCCCTAT

ATATGCTGTCTACAAGAGACCCACCTCAAAACTAGGGACACACACAGGCTGAAAGTGAAAGGATGGAAAA

ACATATTCCATGCAAACGGAGACCAAAAGAAAGCAGGAGTCGCAATACTCATTTCAGATAAAATAGACTT

TAAAATAAAGGATGTGAAAAGGGACAAAGAAGGACACTACATAAAGATCAAAGGATCAATTCAAGAAGAA

GACGTAACAATCATAAATATATATGCACCCAACATAGGAGCACCACAATATATAAGGCAAATACTAACAA

GTATGAAAGGGGAAATTAACAATAACACAATAATAGTGGGAGACTTTAATACTCCACTCACGCATATGGA

TAGATCAACTAAACAGAAAATTAACAAGGAAACACAAACTTTAAATGATACAATAGACAAATTAGATTTA

ATTGATATTTACAGGAGTTTCCACCCCAAAACAATCAATTTTACCTTTTTCTCAAGTGCACATGGAACTT

TCTCCAGGATAGATCACATCCTGGGCCATAAATCTAGCCTTGGTAAATTCAAAAAAATTGAAATCATCCC

AGCCATCTTCTCTGACCACAATGCAGTAAGATTAGACCTCAATTACAGGAAAAAAACTATTAAAAGTTCC

AACATATGGAGGCTAAACAACACGCTTCTGAATAACCAACAAATCACAGAAGAAATCAAAAAAGAAATAA

AATTATGCATAGAAACGAATGAAAATGAAAACACAACAACCCAAAACCTATGGGACACTGTAAAAGCAAT

ACTAAGGGGAAGGTTCATAGCAATACAGGCGTACCTCAAGAAACAAGAAAAAAATCAAATAAATCAACTA

ACTCTACACCTAAAGCAACTAGAGAAGGAAGAAATGAAAAACCCCAGGGTCAGTAGAAGGAAAGAAATCT

TAAAAATTAGGGCAGAAATAAATGCAAAAGAAACAAAAGAGACCATAGCAAAAATCAACAAAACTAAAAG

CTGGTTTTTCGAGAAGATAAACAAAATTGATAAGCCATTAGCCAGACTCATCAAGAAACAAAGGGAGAAG

AATCAGATCAACAAAATTAGAAATGAAAATGGAGAGATCACAACAGACTCCACAGAAATACAAAGGATCA

TAAGAGACTACTACCAACAGCTATATGCCAATAAAATGGACAACTTGGAAGAAATGGACAAATTCTTAGA

AAAGTATAACTTTCCAAAACTGAACCAGGAAGAAATAGAAGATCTTAACAGACCCATCACAAGCACGGAA

ATCGAAACTGTAATCAGAAATCTTCCAGCAAACAAAAGCCCAGGACCAGACGGCTTCACAGCGGAATTCT

ACCAAAAATTTAGAGAAGAGCTAACACCTATCCTACTCAAACTCTTCCAGAAAATTGCAGAAGATGGTAA

ACTTCCAAACTCATTCTATGAGGCCACCATCACCCTAATTCCAAAACCAGACAAAGATGTCACAAAAAAG

GAAAACTACAGGCCAATATCACTGATGAACATAGATGCAAAAATCCTTAATAAAATTCTAGCAAACAGAA

TCCAACATCATATTAAAAAGATCATACATCATGACCAAGTGGGCTTTATCCCAGGAATGCAAGGTTTTTT

TAACATCCGCAAATCAATCAATGTAATACACCACATTAACAAATTGAAAGAAAAAAACCATATGGTTATC

TCAATAGATGCAGAAAAAGCCTTTGACAAAATTCAGCATCCATTTATGATTAAAACCCTCCAGAAAGCAG

GAATAGAAGGAACATATCTCAACATAATAAAAGCTATATATGACAAACCCACAGCAAACATTATCCTCAA

TGGTGAAAAATTGAAAGCATTTCCCCTAAAGTCAGGAACAAGGCAAGGATGCCCACTTTCACCACTACTA

TTCAACATAGTTTTGGAAGTTTTGGCCACAGCAATCAGAGCTGAAAAAGAAATAAAAGGAATACAGATAG

GAAAAGAAGAAGTAAAACTCTCACTGTTTGCAGATGACATGATCCTCTACATAGAAAACCCTAAAGACTC

TACTAGAAAATTACTAGAGTTGATCAATGAATATAGTAAAGTTGCAGGATATAAAATTAACACACAGAAA

TCCCTTGCATTCCTATACTCCAATAATGAGAAAACAGAAAGAGAAATTAAGGAAACAATACCATTCACCA

TTGCATCAAAAAGAATAAAATACTTAGGAATATATCTACCTAAAGAAACAAAAGACTTATATATAGAAAA

CTATAAAACACTGATGAAAGAAATCAAAGAGGACACAAACAGATGGAGAAATATACCGTGTTCATGGATT

GGAAGAATTAATATAGTGAAAATGAGTATACTACCCAAAGCAATCTATAGATTCAATGCAATCCCTATCA

AGCTACCAATGGTATTTTTCACAGAACTAGAACAAATCATCTCACAATTTGTATGGAAATATAAGAAACC

TCGAATAGCCAAAGCAATCTTGAGGAAGAAGAATGGAACTGGAGGAATCAACCTGCCTGACTTCAGGCTC

TACTACAAAGCCACAGTCATCAAGACAGTATGGTACTGGCACAAAGATAGAAATATAGATCAATGGAACA

AAATAGAAAGCCCAGAGATAAACCCACGCACATATGGACACCTTATCTTCGACAAAGGAGGCAAGAATAT

ACAATGGAGAAAAGACAATCTCTTTAACAAGTGGTGCTGGGAAAACTGGTCAACCACTTGTAAAAGAATG

AAACTAGAACACTTCCTAACACCGTACACAAAAATAAACTCAAAATGGATTAAAGATCTAAACGTAAGAC

CAGAAACTATAAAACTCCTAGAGGAAAACATAGGCAAAACACTCTCCGACATAAATCACAGCAGGATCCT

CCATGACCCACCTCCCAGAATATTGGAAATAAAAGCAAAAATAAACAAATGGGACCTAATTAAAATTAAA

AGCTTTTGCACAACAAAGGAAACTATAAGCAAGGTGAAAAGACAGCCTTCAGAATGGGAGAAAATAATAG

CAAATGAAGCAACAGACAAACAATTAATCTCAAAAATATACAAGCAACTTCTGCAGCTGAATTCCAGAAA

AATAAACGACCCAATCAAAAAATGGGCCAAAGAACTAAACAGACATTTCTCCAAAGAAGACATACAGATG

GCTAACAAACACATGAAAAGATGCTCAACATCACTCATTATTAGAGAAATGCAAATCAAAACCACAATGA

GGTATCATTTCACACCAGTCAGAATGGCTGCTATCCAAAAGTCTACAAGCAATAAATGCTGGAGAGGGTG

TGGAGAAAAGGGAACCCTCTTACACTGTTGGTGGGAATGCAAACTAGTACAGCCACTATGGAGAACAGTG

TGGAGATTCCTTAAAAAACTGGGAATAGAACTGCCATATGACCCAGCAATCCCACTGCTGGGCATATACA

CCGAGGAAACCAGAATTGAAAGAGACGCGTGTACCCCAATGTTCATCGCAGCACTGTTTACAATAGCCAG

GACATGGAAGCAACCTAGATGTCCATCAGCAGACGAATGGATAAGGAAGCTGTGGTACATATACACAATG

GAATATTACTCAGCCATTAAAAAAAATACATTTGAATCGGTTCTACTGAGATGGATGAAACTGGAGCCCA

TTATACAGAGTGAAGTAAGCCAGAAAGAAAAACAACAATACAGTATACTAATGCATATATATGGAATTTG

AAAGATGGTAACGATAACCCTATATGCGAGACAGAATAAGAGACACAGATGCATGGAACAATTTTATGGA

CTCTGAGGGAGAAGGTGAGGGTGGGACGTTCTGGGAGAATAGCATTGAAACATGTATATTATCATATGTG

AAACAGAACACCATTCCATGTTCGATGCATGAGACAAGGGCTCAGGGCCGGGGCAATGGGATGACCCAGA

AGGATGGGATGGGGAGGGAGGTGGGTGGGGGGTCCAGGAAGGGGAACACATGTAAACCCATGGCTGATTC

ATGTCAATGTATGGCAAAAACTATTACAATATTGTAAAGTAATTAGCCCCCAACTAAAATAAATAAATTT

ATAAAAAAAAAAAAAAA

**>L1-1A_Gir**

GAGGCGGGGCTAAGATGGCGGAGGAATAGGACGGGGAGACCACTCTCTCCTCCACAAATTTATCGAAAGA

ACATCTGAACGCTGACCAAACTTCACAAAACTACTTCTGAAGGCTGGCAGAGGACGTCAGGCTTCCACAA

AAACTGCGCATTATCTTCAAAAGAGTCCTCTATTACACCTTTAACTTTCATTTTCATAACCCACTATTAA

CTTGCAAAAAAAAAAAAAAACCCTATTTTTGAAGCAAACTACATACATATATCTTATACCTTTTGTGTTT

TCTTTTTTTTAATGTTTTTTTCCTGTTCTACATTTTTTTACTGTTTCTTTTTTTACTGTTTTACTGTTTT

TTTTCTTTTTACACTGTTTCCTTTTTTCTTTTTTTTTTTAGATTGTAAGTTTTATAAGAGTCTAACCTCT

ACTCTAGATTTTTAATCTTTGTTTTACATATTTGATATCAACTTTGTACATTTAAGAACCCAATCTTTAG

GACCCATTTTTACTCAGGAGTGTGATCACTGGCCTGATCACTCTCTCCCCCTATTGATGCTCTTTCTTCT

CCCCCAGGGAACCTCTATCTCCCACCCTCCTCCTTCTCTTCTCAACCCAATTCTGTGAATCTCTGAGGGT

GTTCTGGGTACCGGAGAACACTTCAGGAACAGGATACTGCCCAGATCTCTCTCTCCCCTCTTGATTCCTC

CCTTTCTACTCATGCTCACCTCTATTACCTTCCTCCCCCCTCTCTATGTATCTCTGTGAACCACTCTGAG

GGTTCCAAACTGTGGAAAGCACAGAGGGAAGTGCTTACTGGCTAGATTGCTCTCTCCACATTTGAATTCC

CCTCTTCCCCTCCTGGTCACCTCAACCCCCCCCCCCCACCTCATCTCTCCATGTAACTCAGTGAACCTTT

CTGGGTATCCCTCACTGTGGAGAATCTTTTCACCATTAACCTAAAAGTTTTATTATTGAGGCTGTATGGA

AGGAGAAGTCTTGAGGCTACTGCAAGAATAAGACTGAAAGCCAGGGGAAACATTAATTAATAAGAGACCA

TCCAATAGCCTCCATAAATTCACTGAAACCAAGCTCCACCCAAGAGACAATAAGTTCCAGAGCAAGACAT

ACCACGCAAATTCTCCAGCAACGCAGGAACATAGACCTGAGCATCAATATACAGGCTGCCCAGAGGCACA

CCAAATCCATAGACATCTCAAAAATTAATACTGGTCACTCCATTACACTCTAGAGAGCAGAAATCCAGCT

CCACCCACCAGAACACCGACACAAGCTCCCCCAACCAGGAAGCCTTGACAAGCCACCTGTCCAACCCCAA

CCGCAGTGAGGAACCTCCACAACAAAGAGGAACTTCATACTACCAGAATACAGAAGGGCCACGCCTAACA

CAGCAATCTAAACAAGATGAAAAGGCAGAGAAATACTCAGCAAGTAAAGGAAAATGAGAAATGCCCACAA

AGCCAAACAAAAGAGGAGGAGATAGGGAATCTATCTGAAAAAGAATTTAGAATAAGGATAATAAAAATGA

TCCAAAATCTTGAAAGCAAAATGGAGTTACAGATAAATAGCCTGGAGACAAAGATTGAGAAGATGCAAGA

AATGTTTATCAAGGACTTAGAAGAAATAAAAAGGAATCAATATAGAATGAATAATGAAATAAAGGAGATA

AAAGACACTCTGGAGGGAACCAACAGTAGAATAACGGAGGCAGAAGATAGGATAAGTGAGGTAGAAGATA

GAATGATGGAAATAAACGAAGCAGAGAGGAAAAAAGAAAAAAGAATCAAAAGAAATGAGGAAAACCTCCG

GGACCTCTGGGACAATATGAAACGCCCCAACATTCGAATCATAGGAGTCCCAGAAGAAGAAGATAAAAGG

AAAGGCCATGAAAAAATACTCGAGGAGATAATAGTTGAGAATTTCCCTAAATTGAGGAAAGAAATAGTCA

CAGAAGTCCAAGAAACCCAGAGAGTTCCAAACAGGATAAACCCAAGGCGAAACACCCCAAGACACATATT

AATCAAATTAACAAAGATCAAACACAAAGAACAAATATTAAAAGCAGCAAGGGAAAAACAACAAATAACA

CACAAGGGGATCCCCATAAGGATAACAGCTGATCTTTCAATAGAAACCCTTCAGGCTAGAAGGGAATGGC

AGGACATACTTAAAGTAATGAAAGAGAATAATCTACAACCCAGATTACTGTACCCAGCAAGGATCTCATT

CAGATATGAAGGAGAATTCAAAAGCTTTACAGACAAGCAAGAGCTGAGGGAATTCAGCACCACCAAACCA

GCTCTCCAGCAAATGCTAAAGGATCTTCTCTAAACAGGAAACACAGAAAGGATGTTTCAACACGAACTCA

AAACAACAAAGTAAATGGCAACGGGATCATACTTATCAATAATTACCTTAAATGTAAATGGGTTGAATGC

CCCAACCAAAAGACAAAGGCTTGCTGAATGGATACAAAAACAAGACCCCTATATATGCTGTCTACAAGAG

ACCCACCTCAAAACTAGGGACACACACAGGCTGAAAGTGAAAGGATGGAAAAACATATTCCATGCAAACG

GAGACCAAAAGAAAGCAGGAGTCGCAATACTCATTTCAGATAAAATAGACTTTAAAATAAAGGATGTGAA

AAGGGACAAAGAAGGACACTACATAAAGATCAAAGGATCAATTCAAGAAGAAGACGTAACAATCATAAAT

ATATATGCACCCAACATAGGAGCACCACAATATATAAGGCAAATACTAACAAGTATGAAAGGGGAAATTA

ACAATAACACAATAATAGTGGGAGACTTTAATACTCCACTCACGCATATGGATAGATCAACTAAACAGAA

AATTAACAAGGAAACACAAACTTTAAATGATACAATAGACAAATTAGATTTAATTGATATTTACAGGAGT

TTCCACCCCAAAACAATCAATTTTACCTTTTTCTCAAGTGCACATGGAACTTTCTCCAGGATAGATCACA

TCCTGGGCCATAAATCTAGCCTTGGTAAATTCAAAAAAATTGAAATCATCCCAGCCATCTTCTCTGACCA

CAATGCAGTAAGATTAGACCTCAATTACAGGAAAAAAACTATTAAAAGTTCCAACATATGGAGGCTAAAC

AACACGCTTCTGAATAACCAACAAATCACAGAAGAAATCAAAAAAGAAATAAAATTATGCATAGAAACGA

ATGAAAATGAAAACACAACAACCCAAAACCTATGGGACACTGTAAAAGCAATACTAAGGGGAAGGTTCAT

AGCAATACAGGCGTACCTCAAGAAACAAGAAAAAAATCAAATAAATCAACTAACTCTACACCTAAAGCAA

CTAGAGAAGGAAGAAATGAAAAACCCCAGGGTCAGTAGAAGGAAAGAAATCTTAAAAATTAGGGCAGAAA

TAAATGCAAAAGAAACAAAAGAGACCATAGCAAAAATCAACAAAACTAAAAGCTGGTTTTTCGAGAAGAT

AAACAAAATTGACAAGCCATTAGCCAGACTCATCAAGAAACAAAGGGAGAAGAATCAGATCAACAAAATT

AGAAATGAAAATGGAGAGATCACAACAGACTCCACAGAAATACAAAGGATCATAAGAGACTACTACCAAC

AGCTATATGCCAATAAAATGGACAACTTGGAAGAAATGGACAAATTCTTAGAAAAGTATAACTTTCCAAA

ACTGAACCAGGAAGAAATAGAAGATCTTAACAGACCCATCACAAGCACGGAAATCGAAACTGTAATCAGA

AATCTTCCAGCAAACAAAAGCCCAGGACCAGACGGCTTCACAGCGGAATTCTACCAAAAATTTAGAGAAG

AGCTAACACCTATCCTACTCAAACTCTTCCAGAAAATTGCAGAAGATGGTAAACTTCCAAACTCATTCTA

TGAGGCCACCATCACCCTAATTCCAAAACCAGACAAAGATGTCACAAAAAAGGAAAACTACAGGCCAATA

TCACTGATGAACATAGATGCAAAAATCCTTAATAAAATTCTAGCAAACAGAATCCAACATCATATTAAAA

AGATCATACATCATGACCAAGTGGGCTTTATCCCAGGAATGCAAGGTTTTTTTAACATCCGCAAATCAAT

CAATGTAATACACCACATTAACAAATTGAAAGAAAAAAACCATATGGTTATCTCAATAGATGCAGAAAAA

GCCTTTGACAAAATTCAGCATCCATTTATGATTAAAACCCTCCAGAAAGCAGGAATAGAAGGAACATATC

TCAACATAATAAAAGCTATATATGACAAACCCACAGCAAACATTATCCTCAATGGTGAAAAATTGAAAGC

ATTTCCCCTAAAGTCAGGAACAAGGCAAGGATGCCCACTTTCACCACTACTATTCAACATAGTTTTGGAA

GTTTTGGCCACAGCAATCAGAGCTGAAAAAGAAATAAAAGGAATACAGATAGGAAAAGAAGAAGTAAAAC

TCTCACTGTTTGCAGATGACATGATCCTCTACATAGAAAACCCTAAAGACTCTACTAGAAAATTACTAGA

GTTGATCAATGAATATAGTAAAGTTGCAGGATATAAAATTAACACACAGAAATCCCTTGCATTCCTATAC

TCCAATAATGAGAAAACAGAAAGAGAAATTAAGGAAACAATACCATTCACCATTGCATCAAAAAGAATAA

AATACTTAGGAATATATCTACCTAAAGAAACAAAAGACTTATATATAGAAAACTATAAAACACTGATGAA

AGAAATCAAAGAGGACACAAACAGATGGAGAAATATACCGTGTTCATGGATTGGAAGAATTAATATAGTG

AAAATGAGTATACTACCCAAAGCAATCTATAGATTCAATGCAATCCCTATCAAGCTACCAATGGTATTTT

TCACAGAACTAGAACAAATCATCTCACAATTTGTATGGAAATATAAGAAACCTCGAATAGCCAAAGCAAT

CTTGAGGAAGAAGAATGGAACTGGAGGAATCAACCTGCCTGACTTCAGGCTCTACTACAAAGCCACAGTC

ATCAAGACAGTATGGTACTGGCACAAAGATAGAAATATAGATCAATGGAACAAAATAGAAAGCCCAGAGA

TAAACCCACGCACATATGGACACCTTATCTTCGACAAAGGAGGCAAGAATATACAATGGAGAAAAGACAA

TCTCTTTAACAAGTGGTGCTGGGAAAACTGGTCAACCACTTGTAAAAGAATGAAACTAGAACACTTCCTA

ACACCGTACACAAAAATAAACTCAAAATGGATTAAAGATCTAAACGTAAGACCAGAAACAATAAAACTCC

TAGAGGAAAACATAGGCAAAACACTCTCCGACATAAATCACAGCAGGATCCTCCATGACCCACCTCCCAG

AATATTGGAAATAAAAGCAAAAATAAACAAATGGGACCTAATTAAAATTAAAAGCTTTTGCACAACAAAG

GAAACTATAAACAAGGTGAAAAGACAGCCTTCAGAATGGGAGAAAATAATAGCAAATGAAGCAACAGACA

AACAATTAATCTCAAAAATATACAAGCAACTTCTGCAGCTGAATTCCCGAAAAATAAACGACCCAATCAA

AAAATGGGCCAAAGAACTAAACAGACATTTCTCCAAAGAAGACATACAGATGGCTAACAAACACATGAAA

AGATGCTCAACATCACTCATTATTAGAGAAATGCAAATCAAAACCACAATGAGGTATCATTTCACACCAG

TCAGAATGGCTGCTATCCAAAAGTCTACAAGCAATAAATGCTGGAGAGGGTGTGGAGAAAAGGGAACCCT

CTTACACTGTTGGTGGGAATGCAAACTAGTACAGCCACTATGGAGAACAGTGTGGAGATTCCTTAAAAAA

CTGGGAATAGAACTGCCATATGACCCAGCAATCCCACTGCTGGGCATATACACCGAGGAAACCAGAATTG

AAAGAGACGCGTGTACCCCAATGTTCATCGCAGCACTGTTTACAATAGCCAGGACATGGAAGCAACCTAG

ATGTCCATCAGCAGACGAATGGATAAGGAAGCTGTGGTACATATACACAATGGAATATTACTCAGCCATT

AAAAAAAATACATTTGAATCGGTTCTACTGAGATGGATGAAACTGGAGCCCATTATACAGAGTGAAGTAA

GCCAGAAAGAAAAACAACAATACAGTATACTAATGCATATATATGGAATTTGAAAGATGGTAACGATAAC

CCTATATGCGAGACAGAATAAGAGACACAGATGCATGGAACAATTTTATGGACTCTGAGGGAGAAGGTGA

GGGTGGGACGTTCTGGGAGAATAGCATTGAAACATGTATATTATCATATGTGAAACAGAACACCATTCCA

TGTTCGATGCATGTGACAAGGGCTCAGGGCCGGGGCAATGGGATGATCCAGAAGGATGGGATGGGGAGGG

AGGTGGGTGGGGGGTCCAGGAAGGGGAACACATGTAAACCCATGGCTGATTCATGTCAATGTATGGCAAA

AACTATTACAATATTGTAAAGTAATTAGCCCCCAACTAAAATAAATAAATTTATAAAAAAAAAAAAAAA

**>L1-1B_Gir**

GGCGGGGCTAAGATGGCGGAGGAATAGGACGGGGAGACCACTCTCTCCTCCACAAATTTATCGAAAGAAC

ATCTGAACGCTGACCAAACTTCACAAAACTACTTCTGAAGGCTGGCTGAGGACGTCAGGCTTCCACAAAA

ACAGCGCATTATCTTCAAAAGAGAGAGAAGAAATCCAGCTCCACCCACCAGAACACTGACACAAGCTCCC

CCAACCAGGAAGCCTTGACAAGCCACCTGTCCAACCCCAACCACAGTGAGGAACCTCCACAATAAAGAGG

AACTTCATACTACCAGAATACAGAAGGGCCACGCCTAACACAGCAATCTAAACAAGATGAAAAGGCAGAG

AAATACTCAGCAAGTAAAGGAAAATGAGAAATGCCCACAAAGCCAAACAAAAGAGGAGGAGATAGGGAAT

CTATCTGAAAAAGAATTTAGAATAATGATAATAAAAATGATCCAAAATCCTGAAAGCAAAATGGAGTTAC

AGATAAATAGCCTGGAGACAAAGATTGAGAAGATGCAAGAAATGTTTATCAAGGACTTAGAAGAAATAAA

AAGGAGTCAATATAGAATGAATAATGAAATAAAGGAGATAAAAGACACTCTGGAGGGAACCAATAGTAGA

ATAACGGAGGCAGAAGATAGGATAAGTGAGGTAGAAGATAGAATGATGGAAATAAATGAAGCAGAGAGGA

AAAAAGAAAAAAGAATCAAAAGAAATGAGGAAAACCTCAGGGACCCCTGGGACAATATGAAACGCCCCAA

CATTCGAATCATAGGAGTCCCAGAAGAAGAAGATAAAAGGAAAGGCCATGAAAAAATACTCGAGGAGATA

ATAGTTGAGAATTTCCCTAAATTGAGGAAAGAAATAGTCACAGAAGTCCAAGAAACCCAGAGAGTTCCAA

ACAGGATAAACCCAAGGCGAAACACCCCAAGACACATATTAATCAAATTAACAAAGATCAAACACAAAGA

ACAAATATTAAAAGCAGCAAGGGAAAAACAACAAATAACACACAAGGGGATCCCCATAAGGATAACAGCT

GATCTTTCAATAGAAACCCTTCAGGCTAGAAGGGAATGGCAGGACATACTTAAAGTAATGAAAGAGAATA

ATCTACAACCCAGATTACTGTACCCAGCAAGGATCTCATTCAGATATGAAGGAGAATTCAAAAGCTTTAC

AGACAAGCAAGAGCTGAGGGAATTCAGCACCACCAAACCAGCTCTCCAGCAAATGCTAAAGGATCTTCTC

TAAACAGGAAACACAGAAAGGATGTTTCAACACGAACTCAAAACAACAAAGTAAATGGCAACGGGATCAT

ACTTATCAATAATTACCTTAAATGTAAATGGGTTGAATGCCCCAACCAAAAGACAAAGGCTTGCTGAATG

GATACAAAAACAAGACCCCTATATATGCTGTCTACAAGAGACCCACCTCAAAACTAGCGACACACACAGG

CTGAAAGTGAAAGGATGGAAAAACATATTCCATGCAAACGGAGACCAAAAGAAAGCAGGAGTCGCAATAC

TCATTTCAGATAAAATAGACTTTAAAATAAAGGATGTGAAAAGGGACAAAGAAGGACACTACATAAAGAT

CAAAGGATCAATTCAAGAAGAAGACGTAACAATCATAAATATATATGCACCCAACATAGGAGCACCACAA

TATATAAGGCAAATACTAACAAGTATGAAAGGGGAAATTAACAATAACACAATAATAGTGGGAGACTTTA

ATACTCCACTCACGCATATGGATAGATCAACTAAACAGAAAATTAACAAGGAAACACAAACTTTAAATCA

TACAATAGACAAATTAGATTTAATTGATATTTACAGGAGTTTCCACCCCAAAACAATCAATTTTACCTTT

TCCTCAAGTGCACATGGAACTTTCTCCAGGATAGATCACATCCTGGGCCATAAATCTAGCCTTGGTAAAT

TCAAAAAAATTGAAATCATCCCAGCCATCTTCTCTGACCACAATGCAGTAAGATTAGACCTCAATTACAG

GAAAAAAACTATTAAAAGTTCCAACATATGGAGGCTAAACAACACGCTTCTGAATAACCAACAAATCACA

GAAGAAATCAAAAAAGAAATAAAATTATGCATAGAAACGAATGAAAAGGAAAACACAACAACCCAAAACC

TATGGGACACTGTAAAAGCAATACTAAGGGGAAGATTTATAGCAATACAGGCGTACCTCAAGAAACAAGA

AAAAAATCAAATAAATCAACTAACTCTACACCTAAAGCAACTAGAGAAGGAAGAAATGAAAAACCCCAGG

GTCAGTAGAAGGAAAGAAATCTTAAAAATTAGGGCAGAAATAAATGCAAAAGAAACAAAAGAGACCATAG

CAAAAATCAACAAAACTAAAAGCTGGTTTTTCGAGAAGATAAACAAAATTGACAAGACATTAGCCAGACT

CATCAAGAAACAAAGGGAGAAGAATCAGATCAACAAAATTAGAAATGAAAATGGAGAGATCACAACAGAC

TCCACAGAAATACAAAGGATCATAAGAGACTACTACCAACAGCTATATGCCAATAAAATGGACAACTTGG

AAGAAATGGACAAATTCTTAGAAAAGTATAACTTTCCAAAACTGAACCAGGAAGAAATAGAAGATCTTAA

CAGACCCATCACAAGCACGGAAATCGAAACTGTAATCAGAAATCTTCCAGCAAACAAAAGCCCAGGACCA

GACGGTTTCACAGCGGAATTCTACCAAAAATTTAGAGAAGAGCTAACACCTATCCTACTCAAACTCTTCC

AGAAAATGGCAGAAGATGGTAAACTTCCAAACTCATTCTATGAGGCCACCATCACCCTAATTCCAAAACC

AGACAAAGAGGTCACAAAAAAGGAAAACTACAGGCCAATATCACTGATGAACATAGATGCAAAAATCCTT

AATAAAATTCTAGCAAACAGAATCCAACATCATATTAAAAAGATCATACATCATGACCAAGTGGGCTTTA

TCCCAGGAATGCAAGGTTTTTTTAACATCCGCAAATCAATCAATGTAATACACCACATTAACAAATTGAA

AGAAAAAAACCATATGGTTATCTCAATAGATGCAGAAAAAGCCTTTGACAAAATTCAGCATCCATTTATG

ATTAAAACCCTCCAGAAAGCAGGAATAGAAGGAACATATCTCAACATAATAAAAGCTATATATGACAAAC

CCACAGCAAACATTATCCTCAATGGTGAAAAATTGAAAGCATTTCCCCTAAAGTCAGGAACAAGGCAAGG

ATGCCCACTTTCACCACTACTATTCAACATAGTATTGGAAGTTTTGGCCACAGCAATCAGAGCTGAAAAA

GAAATAAAAGGAATACAGATAGGAAAAGAAGAAGTAAAACTCTCACTGTTTGCAGATGACATGATCCTCT

ACATAGAAAACCCTAAAGACTCTACTAGAAAATTACTAGAGTTGATCAATGAATATAGTAAAGTTGCAGG

ATATAAAATTAACACACAGAAATCCCTTGCATTCCTATACTCCAATATTGAGAAAACAGAAAGAGAAATT

AAGGAAACAATACCATTCACCATTGCATCAAAAAGAATAAAATACTTAGGAATATATCTACCTAAAGAAA

CAAAAGACTTATATATAGAAAACTATAAAACACTGATGAAAGAAATCAAAGAGGACACAAACAGATGGAG

AAATATACCGTGTTCATGGATTGGAAGAATTAATATAGTGAAAATGAGTATACTACCCAAAGCAATCTAT

AGATTCAATGCAATCCCTATCAAGCTACCAATGGTATTTTTCACAGAACTAGAACAAATCATCTCACAAT

TTGTATGGAAATATAAGAAACCTCGAATAGCCAAAGCAATCTTGAGGAAGAAGAATGGAACTGGAGGAAT

CAACCTGCCTGACTTCAGGCTCTACTACAAAGCCACAGTCATCAAGACAGTATGGTACTGGCACAAAGAT

AGAAATATAGATCAATGGAACAAAATAGAAAGCCCAGAGATAAACCCACGCACATATGGACACCTTATCT

TCGACAAAGGAGGCAAGAATATACAATGGAGAAAAGACAATCTCTTTAACAAGTGGTGCTGGGAAAACTG

GTCAACCACTTGTAAAAGAATGAAACTAGAACACTTCCTAACACCATACACAAAAATAAACTCAAAATGG

ATTAAAGATCTAAACGTAAGACCAGAAACAATAAAACTCCTAGAGGAAAACATAGGCAAAACACTCTCCG

ACATAAATCACAGCAGGATCCTCCATGACCCACCTCCCAGAATATTGGAAATAAAAGCAAAAATAAACAA

ATGGGACCTAATTAAAATTAAAAGCTTTTGCACAACAAAGGAAACTATAAGCAAGGTGAAAAGACAGCCT

TCAGAATGGGAGAAAATAATAGCAAACGAAGCAACAGACAAACAATTAATCTCAAAAATATACAAGCAAC

TTCTGCAGCTGAATTCCAGAAAAATAAACGACCCAATCAAAAAATGGGCCAAAGAACTAAACAGACATTT

CTCCAAAGAAGACATACAGATGGCTAACAAACACATGAAAAGATGCTCAACATCACTCATTATTAGAGAA

ATGCAAATCAAAACCACAATGAGGTATCATTTCACACCAGTCAGAATGGCTGCTATCCAAAAGTCTACAA

GCAATAAATGCTGGAGAGGGTGTGGAGAAAAGGGAACCCTCTTACACTGTTGGTGGGAATGCAAACTAGT

ACAGCCACTATGGAGCACAGTGTGGAGATTCCTTAAAAAACTGGGAATAGAACTGCCATATGACCCAGCA

ATCCCACTGCTGGGCATATACACCGAGGAAACCAGAATTGAAAGAGACGCGTGTACCCCAATGTTCATCG

CAGCACTGTTTACAATAGCCAGGACATGGAAGCAACCTAGATGTCCATCAGCAGACGAATGGATAAGGAA

GCTGTGGTACATATACACAATGGAATATTACTCAGCCATTAAAAAAAATACATTTGAATCGGTTCTACTG

AGATGGATGAAACTGGAGCCCATTATACAGAGTGAAGTAAGCCAGAAAGAAAAACAACAATACAGTATAC

TAATGCATATATATGGAATTTGAAAGATGGTAACGATAACCCTATATGCGAGACAGAATAACAGACACAG

ATGCATGGAACAATTTTATGGACTCTGAGGGAGAAGGTGAGGGTGGGACGTTCTGGGAGAATAGCATTGA

AACATGTATATTATCATATGTGAAACAGAACACCATTCCATGTTCGATGCATGAGACAAGGGCTCAGGGC

CGGGGCAATGGGATGACCCAGAGGGATGGGATGGGGAGGGAGGTGGGTGGGGGGTCCAGGAAGGGGAACA

CATGTAAACCCATGGCTGATTCATGTCAATGTATGGCAAAAACTATTACAATATTGTAAAGTAATTAGCC

CCCAACTAAAATAAACAAATTTATAAAAAAAAAA

**SINE elements**

**>Bov-tA1_Gir**

GGGCTTCCCAGGTGGCGCTAGTGGTAAAGAACCCGCCTGCCAATGCAGGAGACGTAAGAGACGCGGGTTC

GATCCCTGGGTCGGGAAGATCCCCTGGAGGAGGGCATGGCAACCCACTCCAGTATTCTTGCCTGGAGAAT

CCCATGGACAGAGGAGCCTGGCGGGCTACAGTCCATAGGGTCGCAAAGAGTCGGACACGACTGAAGCGAC

TTAGCACACACACA

**>Bov-tA2a_Gir**

GGGCTTCCCTGGTGGCTCAGAGGTTAAAGCATCTGCCTCCAATGCGGGAGACCCGGGTTCGATCCCTGGG

TTGGGAAGATCCCCTGGAGAAGGAAATGGCAACCCACTCCAGTATTCTTGCCTGGAGAATCCCATGGACG

GAGGAGCCTGGTGGGCTACAGTCCATGGGGTCGCAAAGAGTCGGACACGACTGAGCGACTTCACTTCACT

TCAC

**>Bov-tA2a1_Gir**

GGGCTTCCCTGGTAGCTCAGATGGTAAAGCGTCTGCCTACAATGCAGGAGACCCGGGTTCGATCCCTGGG

TTGGGAAGATCCCCTGGAGAAGGAAATGGCAACCCACTCCAGTATTCTTGCCTGGAGAATTCCATGGACC

GAGGAGCCTGGTGGGCTACAGTCCATGGGGTCGCAAAGAGTCGGACACGACTGAGCAACTTCACTTCACT

TCAC

**>Bov-tA2b_Gir**

GGGCTTCCCTGGTGGCTCAGATGGTAAAGAATCTGCCTGCAATGCAGGAGACCTGGGTTCGATCCCTGGG

TTGGGAAGATCCCCTGGAGAAGGGAATGGCAACCCACTCCAGTATTCTTGCCTGGAGAATCCCATGGACA

GAGGAGCCTGGTGGGCTACAGTCCATGGGGTCGCAAAGAGTCGGACACGACTGAGCGACTAACACTTTCA

CTTT

**>Bov-tA2c_Gir**

GGGCTTCCCTTGTAGCTCAGTTGGTAAAGAATCTGCCTGCAGTGCAGGAGACCTGGGTTCGATCCCTGGG

TTGGGAAGATCCCCTGGAGAAGGAAATGGCAACCCACTCCAGTATCCTTGCCTGGAAAATCTCATGGACA

GAGGAGCCTGGTGGGCTGCAGTCCATGGGGTCGCAAAGAGTCGGGCACGACTGAGCGACTAACACTTACT

TA

**>Bov-tA2d_Gir**

GGGCTTCCCTGGTGGCTCAGATGGTAAAGAATCTGCCTGCAATGCAGGAGACCTGGGTTCGATCCCTGGG

TCGGGAAGATCCCCTGGAGAAGGAAATGGCAACCCACTCCAGTATTCTTGCCTGGAGAATCCCATGGACA

GAGGAGCCTGGCGGGCTACAGTCCATGGGGTCGCAAAGAGTCGGACACGACTGAGCGACTAACACACACA

**>Bov-tA2e_Gir**

GGGCTTCCCTCGTAGCTCAGTTGGTAAAGAATCTGCCTGCAATGCAGGAGACCCGGGTTCGATTCCTGGG

TCGGGAAGATCCCCTGGAGAAGGAAATGGCAACCCACTCCAGTATTCTTGCCTGGAGAATCCCATGGACA

GAGGAGCCTGGCGGGCTACAGTCCATGGGGTCGCAAGAGTCGGACACGACTTAGCGACTAAACCACCACC

ACCACC

**>Bov-tA3_Gir**

GGACTTCCCTGGTGGCTCAGAGGTTAAAGCGTCTGCCTCCAATGCGGGAGACCTGGGTTCGATCCCTGGG

TCGGGAAGATCCCCTGGAGAAGGAAATGGCAAACCACTCCAGTATTCTTGCCTGGAGAATCCCATGGACG

GAGGAGCCTGGCAGGCTACAGTCCACGGGGTCGCAAAGAGTCGGACACGACTGAGCGACTTTCCTTTC

**>Bov-tA3a_Gir**

AGACTTCCCTGGTGGCTCATACGGTAAAGCGTCTGCCTCCAATGCGGGAGACCCGGGTTCAATCCCTGGG

TCGGGAAGATCTCCTGGAGAAGGAAATGGCAACCCACTCCAGTATTCTTGCCTGGAAAATCCCATGGACA

GAGGAGCCTGGCAGGCTACAGTCCATGGGGTCGCAAAGAGTCGGACACGACTGAGCAACTTCACTTCACT

TCA

**>Bov-A2_Gir**

GGAGAAGGCAATGGCACCCCACTCCAGTACTCTTGCCTGGAAAATCCCATGGACGGAGGAGCCTGGTAGG

CTACAGTCCATGGGGTCGCTAAGAGTCGGACACGACTGAGCGACTTCACTTTCACTTTTCCCTTTCATGC

ACTGGAGAAGGAAATGGCAACCCACTCCAGTATTCTTGCCTGGAGAATCCCAGGGACGGCGGAGCCTGGT

GGGCTGCCGTCTATGGGGTCGCACAGAGTCGGACACGACTGAAGCGACTTAGCAGCAGC

**>Bov-A2b_Gir**

GGAGAAGGCAATGGCACCCCACTCCAGTACTCTTGCCTGGAAAATCCCATGAACGGAGGAGCCTGGCGGG

CTGCAGTCCATGGGGTCACTGAGTCGGACACGACTGAGCGACTCCACTTTCACTTTTCACTTTCATGCCC

TGGAGAAGGAAATGGCAACCCACTCCAGTACTCTTGCCTGGAGAATCCCAGGGACGGGGGAGCCTGGTGG

GCTGCCGTCTATGGGGTCGCACAGAGTCGGACACGACTGACGCGACTTAGCAGCAGC

**>Bov-A2c_Gir**

GGAGAAGGCAATGGCACCCCACTCCAGTACTCTTGCCTGGAAAATCCCATGGACAGAAGAGCCTGGTAGG

CTGCAGTCCATGGGGTCGCGAAGAGTCAGACACGACTGAGCGACTTCACTTTCACTTTTCACTTTCATGC

ATTGGAGAAGGAAATGGCAACCCACTCCAGTGTTCTTGCCTGGAGAATCCCAGGGACAGCGGAGCCTGGT

GGGCTTCCGTCTATGGGGTCGCACAGAGTCGGACACGACTGAAGCGACGCAGCAGCAGC

**>Bov-A2d_Gir**

GGAGAAGGAAATGGCAACCCACTCCAGTACTCTTGCCTGGAAAATCCTATGGACAGAGGAGCCTGGTAGG

CTGCAGTCCGTGGGTTCGCTAAGGGTCGGACACGACTAAGCGACTTCACTTTCACTTTTCCCTTTCATGC

ACTGGAGAAGGAAATGGCAACCCACTCCAGTATTCTTGCCTGGAGAATCCTAGGGACGGCAGAGCCTGGT

GGGCTGCCGTCTATAGGGTCGCACAGAGTCGGACACGACTGAAGCGACTTAGCAGTAGC

**>Bov-tA-monoA_Gir**

TGGAGAAGGAAATGGCAACCCACTCCAGTATTCTTGCCTGGAGAATCCCATGGACAAAGGAGCCTGGTGG

GCTACAGTCCATGGGGTCGCAAAGAGTCGGACACGACTGAGCGACTAACACACACACA

**>Bov-tA-monoB_Gir**

TGGAGAAGGAAATGGCAACCCACTCCAGTATTCTTGCCTGGAGAATCCCAGGGACAAAGGAGCCTGGTGG

GCTACAGTCCATGGGGTCACAAAGAGTCGGACACGACTGAGTGACTAACACACACACA

**>CHR-2_Gir**

GGGCTTCCCTGGTGGCTCAGTGGTAAAGAATCCGCCTGCCAATGCAGGAGACATGGGTTCGATCCCTGAT

CTGGGAAGATCCCACATGCCACGGAGCAACTAAGCCCGTGCGCCACAACTACTGAGCCTGTGCTCTAGAG

CCCGGGAGCTGCAACTACTGAGCCCATGTGCCGCAACTACTGAAGCCCGCGCGCCCTAGAGCCCGTGCTC

CGCAACAAGAGAAGCCACCGCAATGAGAAGCCCGCGCACCACAACTAGAGAGTAGCCCCTGCTCGCCGCA

ACTAGAGAAAAGCCCGCGCAGCAACGAAGACCCAGCACAGCCAAAAA
