# Supplementary material for: Population analysis of retrotransposons in giraffe genomes supports RTE decline and widespread LINE1 activity in Giraffidae
Source: Mob DNA. 2021 Nov 26;12:27. doi: 10.1186/s13100-021-00254-y (PMC8620236; doi:10.1186/s13100-021-00254-y)
Supplement: Supplementary file 3 — Additional file 3. Results of the clustering analysis of SINE activity in the Kordofan giraffe genome. [file 13100_2021_254_MOESM3_ESM.docx]

Supplemental data set 2

**Sequences of 85 Bov-A2_Gir elements representing 85 clusters composed of identical Bov-A2 elements, where Bov-A2.N1.N2 stays for the Bov-A2 cluster number N1, which is composed of N2 identical sequences (all segmental duplications have been excluded):**

>Bov-A2.1.15

TGGAGAAGGCAATGGCACCCCACTCCAGTACTCTTGCCTGGAAAATCCCATGAACGGAGGAGCCTGGCGG

GCTGCAGTCCATGGGGTCACTGAGTCGGACACGACTGAGCGACTCCACTTTCACTTTTCACTTTCATGCC

CTGGAGAAGGAAATGGCAACCCACTCCAGTACTCTTGCCTGGAGAATCCCAGGGACGGGGGAGCCTGGTG

GGCTGCCGTCTATGGGGTCGCACAGAGTCGGACACGACTGACGCGACTTAGCAGCAGCAGCAGCAGC

>Bov-A2.2.14

CGGAGAAGGCAATGGCACCCCACTCCAGTACTCTTGCCTGGAAAATCCCATGGACGGAGGAGCCTGGTAG

GCTACAGTCCATGGGGTCGCTAAGAGTCGGACACGACTGAGCGACTTCACTTTCACTTTTCCCTTTCATG

CACTGGAGAAAGAAATGGCAACCCACTCCAGTATTCTTGCCTGGAGAATCCTAGGGACGGCAGAGCCTGG

TGAGCTGCCGTCTATGGGGTCGCACAGAGTCGGACACGACTGAAGCGACTTAGCATAGCATAGCATAGCA

T

>Bov-A2.3.13

CGGAGAAGGCAATGGCACCCCACTCCAGTACTCTTGCCTGGAAAATCCCATGGACGGAGGAGCCTGGTAG

GCTACAGTCCATGGGGTCGCTAAGAGTCGGACACGACTGAGCGACTTCACTTTCACTTTTCCCTTTCATG

CACTGGAGAAAGAAATGGCAACCCACTCCAGTATTCTTGCCTGGAGAATCCTAGGGACGGCGGAGCCTGG

TGAGCTGCCGTCTATGGGGTCGCACAGAGTCGGACACGACTGAAGCGACTTAGCATAGCATAGCATAGCA

T

>Bov-A2.4.8

TGGAGAAGGCAATGGCACCCCACTCCAGTACTCTTGCCTGGAAAATCCCATGGATGGAGGAGCCTGGTAG

GCCACAGTCCATGGGGTCGCTAAGAGTCGGACACGACTGAGCGACTTCACTTTCACTTTTCCCTTTCATG

CACTGGAGAAGGAAATGGCAACCCACTCCAGTATTCTTGCCTGGAGAATCCCAGGGACGTCGGAGCCCGG

TGGCTGCCGTCTATGGGGTCGCACAGAGTCGGACACGACTGAAGCGACTTAGCAGCAGCAGCAGCAGC

>Bov-A2.5.8

CGGAGAAGGCAATGGCACCCCACTCCAGTACTCTTGCCTAGAAAATCCCATGGATGGAGGAGCCTGGTAG

GCTACAGTCCATGGGGTCGCTACGAGTCGGACACGACTGAGCAACTTCACTTTCACTTTTCCCTTTCATG

CACTGGAGAAGGAAATGGCAACCCACTCCAGTATTCTTGCCTGGAGAATCCTAGGGACGGCGGAGCCTGG

TGAGCTGCCGTCCATGGGGTCGCACAGAGTCGGACACGACTGAAGCGACTTAGCAGCAGCAGCAGCAGC

>Bov-A2.6.6

CGGAGAAGGCAATGGCACCCCACTCCAGTACTCTTGCCTGGAAAATCCCATGGACGGAGGAGCCTGGTAG

GCTACAGTCCATGGGGTCGCTAAGAGTCGGACACGACTGAGCGACTTCACTTTCACTTTTCCCTTTCATG

CACTGGAGAAGGAAATGGCAACCCACTCCAGTATTCTTGCCTGGAGAATCCCAGGGACGGCGGAGCCTGG

TGGGCTGCCGTCTATGGGGTCGCACAGAGTCGGACACGACTGAAGCGACTTAGCAGCAGCAGC

>Bov-A2.7.5

GGGAGAAGGCAATGGCACCCCACTCCAGTACTCTTGCCTGGAAAATCCCATGGACAGAAGAGCCTGGTAG

GCTGCAGTCCATGGGGTCGCGAAGAGTCAGACACGACTGAGCGACTTCACTTTCACTTTTCACTTTCATG

CATTGGAGAAGGAAATGGCAACCCACTCCAGTGTTCTTGCCTGGAGAATCCCAGGGACAGCGGAGCCTGG

TGGGCTTCCGTCTATGGGGTCGCACAGAGTCGGACACGACTGAAGCGACGCAGCAGCAGCAGCAGC

>Bov-A2.8.5

CGGAGAAGGCAATGGCACCCCACTCCAGTACTCTTGCCTGGAAAATCCCATGGACGGAGGAGCCTAGTAG

GCTGCAGTCTATGGGGTCGCTAGGGGTCGGACACGACTGAGCGGCTTCACTTTCACTTTTCACTTTCATG

CATTGGAGAAGGAAATGGCAGCCCACTCCAGTATTCTTGCCTGGAGAATCCCAGGGACGGGGGAGCCCGG

TGGGCTGCCGTCTATGGGGTCGCACAGAGTCGGACACGACTGAAGCGACTTAGCAGCAGCAGCAGC

>Bov-A2.9.4

CGGAGAAGGCAATGGCACCCCACTCCAGTACTCTTGCCTGGAAAATCCCATGGACGGAGGAGCCTGGTAG

GCTGTAGTCCATGGGGTCGCTAAGAGTCAGACATGACTGAGCGACTTCACTTTCACTTTTCCTGTTCATG

CACTGGAGAAGGAAATGGCAACCCACTCCAGTATTCTTGCCTGGAGAATCCCAGGGATGGCGGAGCCTGG

TGGGCTGCCGTCTATGGGGTCGCACAGAGTCGGACACGACTGAAGCGACTTAGCAGCAGCAGCAGCAGCA

GC

>Bov-A2.10.4

CGGAGAAGGCAATGGCACCCCACTCCAGTACTCTTGCCTGGAAAATCCCATGGACGGAGGAGCCTGGTAG

GCTACAGTCCATGGGGTCGCTAAGAGTCGGACGACTGAGCGACTTCACTTTCACTTTTCCCTTTCATGCA

CTGGAGAAGGAAACGGCAACCCACTCCAGTATTCTTGCCTGGAGAATCCCAGGGACGGCGGAGCCTGGTG

GGCTGCCGTCTATGGGGTCGCACAGAGTCGGACACGACTGAAGCGACTTAGCAGCAGCAGCAGCAGC

>Bov-A2.11.3

CGGAGAAGGCAATGGCACCCCACTCCAGTCCTCTTGCCTGGAAAATCCCATGGATGGAGGAGCCTGGTAG

GCTACAGTCCATGGGGTCGCTAAGAGTCGGACACGACTGAGCGACTTCACTTTCACTTTTCCCTTTCATG

CACTGGAGAAGGAAATGGCAACCCACTCCAGTATTCTTGCCTGGAGAATCCCAGGGACGGCGGAGCCTGG

TGGGCTGCCGTCTATGGGGTCGCACAGAGTCGGACACGACTGAAGCGACTTAGCAGCAGCAGCAGCAGC

>Bov-A2.12.3

CGGAGAAGGCAATGGCACCCCACTCCAGTACTCTTGCCTGGAAAATCCCATGGACAGAGGAGCCTGGTAG

GCTACAGTCCATGGGGTCGCTAAGAGTCGGACACGACTGAGCGACTTCACTTTCACTTTTCCCTTTCATG

CACTGGAGAAAGAAATGGCAACCCACTCCAGTATTCTTGCCTGGAGAATCCTAGGGACGGCGGAGCCTGG

TGAGCTGCCGTCTATGGGGTCGCACAGAGTCGGACACGACTGAAGCGACTTAGCATAGCATAGCATAGCA

T

>Bov-A2.13.3

CGGAGAAGGCAATGGCACCCCACTCCAGTACTCTTGCCTGGAAAATCCCATGGACGGAGGAGCCTGGTAG

GCTACAGTCCATGGGGTCGCTAAGAGTCGGACACGACTGAGCGACTTCACTTTCACTTTTCACTCTCATG

CATTGGAGAAGGAAATGGCAACCCACTCCAGTATTCTTGCCTGGAGAATCCTAGGGACGGCGGAGCCTGG

TGAGCTGCCGTCTATGGGGTCGCACAGAGTCGGACACGACTGAAGCGACTTAGCATAGCATAGCATAGCA

T

>Bov-A2.14.3

CGGAGAAGGCAATGGCACCCCACTCCAGTACTCTTGCCTGGAAAATCCCATGGACAGAGGAGCCTGGTAG

GCTACAGTCCATGGGGTCGCTAAGAGTCGGACACGACTGAGCGACTTCACTTTCACTTTTCCCTTTCATG

CACTGGAGAAAGAAATGGCAACCCACTCCAGTATTCTTGCCTGGAGAATCCTAGGGACGGCAGAGCCTGG

TGAGCTGCCGTCTATGGGGTCGCACAGAGTCGGACACGACTGAAGCGACTTAGCATAGCATAGCATAGCA

T

>Bov-A2.15.3

CGGAGAAGGCAATGGCACCCCACTCCAGTACTCTTGCCTGGAAAATCCCATGGACGGAGGAGCCTGGTAG

GCTGCAGTCCATGGGGTCGCTAAGAGTCGGACACGACTGAGCGACTTCACTTTCACTTTTCCCTTTCATG

CACTGGAGAAGGAAATGGCAACCCACTCCAGTATTCTTGCCTGGAGAATCCCAGGGACGGCGGAGCCTGG

TGGGCTGCCGTCTATGGGGTCGCACAGAGTCGGACACGACTGAAGCGACTTAGCAGCAGCAGCAGCAGCA

GCAGC

>Bov-A2.16.3

CGGAGAAGGCAATGGCACCCCACTCCAGTACTCTTGCCTGGAAAATCCCATGGATGGAGGAGCCTGGTAG

GCTGCAGTCCATGGGGTCGCTAAGAGTCGGACACGACTGAGCGACTTCACTTTCACTTTTCCCTTTCATG

CACTGGAGAAGGAAATGGCAACCCACTCCAGTATTCTTGCCTGGAGAATCCCAGGGACGGCGGAGCCTGG

TGGGCTGCCGTCTATGGGGTCGCACAGAGTCGGACACGACTGAAGCGACTTAGCAGCAGCAGCAGCAGCA

GC

>Bov-A2.17.3

CGGAGAAGGCAATGGCACCCCACTCCAGTACTCTTGCCTGGGAAACCCCATGGACAGAGGAGCCTGGTAG

GCTGCAGCCCATGGGGTCGCGGAGAGTCGGACACGACTGAGCGACTTCACTTTCACTTTTCACTGTCATG

CACTGGAGGAGGAAATGGCAACCCACTCCAGTATTCTTGCCTGGAGAATCCCAGGGACGGGGGAGCCTGG

AGGGCTGCCGTCTATGGGGTCGCACAGAGTCGGACACGACTGACGCGACTAAGCAGCAGCAGCAGCAGCA

GC

>Bov-A2.18.3

CGGAGAAGGCAATGGCACCCCACTCCAGTACTCTTGCCTGGACAATCCCATGGACGGAGGAGCCTGGTAG

GCTACAGTCCATGGGGTCGCTAAGAGTCGGACACGACTGAGCGACTTCACTTTCACTTTTCACTTTCATG

CACTGGAGAAGGAAATGGCAGCCCACTCCAGTATTCTTGCCTGGAGAATCCCAGGGACGGCGGAGCCTGG

TGGGCTGCCGTCTATGGGGTCGCACAGAGTCGGACACGACTGAAGCGACTTAGCAGCAGCAGCAGCAGC

>Bov-A2.19.3

CGGAGAAGGCAATGGCACCCCACTCCAGTACTCTTGCCTGGAAAATCCCATGGATGGAGGAGCCTGGTAG

GCTACAGTCCATGGGGTCGCTAAGAGTCGGACACGACTGAGCGACTTCACTTTCACTTTTCCCTTTCATG

CACTGGAGAAGGAAATGGCAACCCACTCCAGTATTCTTGCCTGGAGAATCCCAGGGATGGCAGAGCCTGG

TGGGCTGCCGTCTATGGGGTCGCACAGAGTCGGACACGACTGAAGCGACTTAGCAGCAGCAGCAGCAGC

>Bov-A2.20.3

CGGAGAAGGCAATGGCACCCCACTCCAGTACTCTTGCCTGGAAAATCCCATGGACGGAGGAGCCTGGTAG

GCTACAGTCCATGGGGTCGCTGAGAGTCGGACACGACTGAGCGACTTCACTTTCACTTTTCCCTTTCATG

CACTGGAGAAGGAAATGGCAACCCACTCCAGTATTCTTGCCTGGAGAATCCCAGGGATGGCGGAGCTTGG

TGGGCTGCCGTCTATGGGGTCGCACAGAGTCGGACACGACTGAAGCGACTTAGCAGCAGCAGCAGC

>Bov-A2.21.3

CGGAGAAGGCAATGGCACCCCACTCCAGTACTCTTGCCTGGAAAATCCCATGGACGGAGGAGCCTAGTAG

GCTACAGTCCATGGGGTCGCTAAGAGTCGGACACGACTGAGCGACTTCACTTTCACTTTACCCTTTCATG

CACTGGAGAAGGAAATGGCAACCCACTCCAGTATTCTTGCCTGGAGAATCCCAGGGATGGCGGAGCCTGG

TGGGCTGCCGTCTATGGGGTCGCACAGAGTCGGACACGACTGAAGCGACTTAGCAGCAGCAGCAGC

>Bov-A2.22.3

CGGAGAAGGCAATGGCACCCCACTCCAGTACTCTTGCCTGGAAAATCCCATGGACAGAGGAGCCTGGTAG

GCTGCAGTCCATGGGGTCGCTAAGAGTCAGACATGACTGAGCGACTTCACTTTCACTTTTCACTTTCATG

CATTGGAGAAGGAAATGGCAACCCACTCCAGTGTTCTTGCCTGGAGAATCCCAGGGACGGGGGAGCCTAG

TGGGCTGCCGTCTATGGGGTCGCACAGAGTCGGACACGACTGAAGCGACTTAGCAGCAGCAGCAGCAGC

>Bov-A2.23.3

TGGAGAAGGCAATGGCACCCCACTCCAGTACTCTTGCCTGGAAAATCCCATGGACGGAGGAGCCTGGTAG

GCTGTAGTCCATGGGGTCGCTAAGAGTCAGACATGACTGAGCGACTTCACTTCACTTTTCCTGTTCATGC

ACTGGAGAAGGAAATGGCAACCCACTCCAGTATTCTTGCCTGGAGAATCCCAGGGATGGCGGAGCCTGGT

GGGCTGCCATCTATGGGGTCGCACAGAGTCGGACACGACTGAAGCGACTTAGCAGCAGCAGCAGC

>Bov-A2.24.3

CGGAGAAGGCAATGGCACCCCACTCCAGTACTCTTGCCTGGAAAATCCCATGGACGGAGGAGCCTGGTAG

GCTACAGTCCATGGGGTCGCTAAGAGTCGGACACGACTGAGCAACTTCACTTTCACTTTTCCCTTTCATG

CACTGGAGAAAGAAATGGCAACCCACTCCAGTATTCTTGCCTGGAGAATCCTAGGGACGGCAGAGCCTGG

TGAGCTGCCGTCTATGGGGTCGCACAGAGTCGGACACGACTGAAGCGACTTAGCATAGCATAGCATAGCA

T

>Bov-A2.25.3

CGGAGAAGGCAATGGCACCCCACTCCAGTACTCTTGCCTGGAAAATCCCATGGACGGAGGAGCCTAGTAG

GCTGCAGTCTATGGGGTCGCTAGGGGTCGGACACGACTGAGCGGCTTCACTTTCACTTTTCACTTTCATG

CATTGGAGAAGGAAATGGCAGCCCACTCCAGTATTCTTGCCTGGAGAATCCCAGGGACGGGGGAGCCTGG

TGGGCTGCCGTCTATGGGGTCGCACAGAGTCGGACACGACTGAAGCGACTTAGCAGCAGCAGCAGC

>Bov-A2.26.3

CGGAGAAGGCAATGGCACCCCACTCCAGTACTCTTTTGCCTGGAAAATCCCATGGATGGAGGAGCCTGGA

AGGCTTCAGTCCATGGGGTCGCTAAGAGTCGGACACGACTGAGCTACTTCACTTTCGCTTTTCCCTTTCA

TGCACTGGAGAAGGAAATGGCAACCCACTCCAGTATTCTTGCCTGGAGAATCCCAGGGATGGCAGAGCCT

GGTGGGCTGCCGTCTATGGGGTCGCACAGAGTCGGACACGACTGAAGCGACTTAGCAGCAGCAGCAGCAG

CAGC

>Bov-A2.27.3

CAGAGAAGGCAATGGCACCCCACTCCAGTACTCTTGCCTGGAAAATCCCATGGACGGAGGAGCCTGGTAG

GCTACAGTCCATGGGGTCGCTAAGAGTCGGACACGACTGAGCGACTTCACTTTCACTTTTCCCTTTCATG

CACTGGAGAAAGAAATGGCAACCCACTCCAGTATTCTTGCCTGGAGAATCCTAGGGACGGCAGAGCCTGG

TGAGCTGCCGTCTATGGGGTCGCACAGAGTCGGACACGACTGAAGCGACTTAGCATAGCATAGCATAGCA

TAGCAT

>Bov-A2.28.2

CGGAGAAGGCAATGGCACCCCACTCCAGTACTCTTGCCTGGAAAATCCCACGGATGGAGGAGCCTGGTAG

GCTACAGTCCATGGGGTCGCTAAGAGTCGGACATGACTGAGCGACTTCACTTTCACTTTTCCCTTTCATG

CACTGGAGAAGGAAATGGCAACCCACTCCAGTATTCTTGCCTGGAGAATCCCAGGGACGGCGGAGCCTGG

TGGGCTGCCATCTATGGGGTCGCACAGAGTCGGACACGACTGAAGCGACTTAGCAGCAGCAGCAGC

>Bov-A2.29.2

GGAGAAGGCAATGGCACCCCACTCCAGTACTCTTGCCTGGAAAATCCCATGGACGGAGGAGCCTGGTAGG

CTACAGTCCATGGGGTCGCTAAGAGTCGGACGACTGAGCGACTTCACTTTCACTTTTCCCTTTCATGCAC

TGGAGAAGGAAACGGCAACCCACTCCAGTATTCTTGCCTGGAGAATCCCAGGGACGGCGGAGCCTGGTGG

GCTGCCGTCTATGGGGTCGCACAGAGTCGGACACGACTGAAGCGACTTAGCAGTAGCAGCAGCAGC

>Bov-A2.30.2

CGGAGAAGGCAATGGCACCCCACTCCAGTACTCTTGCCTGGAAAATCCCATGGATGGAGGAGCCTGGTAG

GCTGCAGTCCATGGGGTCGCTAAGAGTCGGACACGACTGAGCGACTTCACTTTCACTTTTCCCTTTCATG

CACTGGAGAAGGAAATGGCAACCCACTCCAGTATTCTTGCCTGGAGAATCCCAGGGACGGCGGAGCCTGG

TGGGCTGCCGTCTATGGGGTCGCACAGGGTCGGACACGACTGAAGCGACTTAGCAGCAGCAGCAGC

>Bov-A2.31.2

CGGAGAAGGCAATGGCACCCCACTCCAGTACTCTTGCCTGGAAAATCCCATGGGTGGAGGAGCCTGGTAG

GCTGCAGACCCATGGGGTCGCTAGGAGTCGGACACGACTGAGCGACTTCACTTTCACTTTTCCCTTTCAT

GCACTGGAGAAGGAAATGGCAACCCACTCCAGTATTCTTGCCTGGAGAATCCCAGGGACGGCGGAGCCTG

GTGGGCTGCCGTCTATGGGGTCGCACAGAGTCGGACACGACTGAAGCGACTTAGCAGCAGCAGCAGC

>Bov-A2.32.2

CGGAGAAGGCAATGGCACCCCACTCCAGTACTCTTGCCTGGAAAATCCCATGGATGGAGGAGCCTTGTAG

GCTACAGTCCATGGGGTCGCTAAGAGTCAGACATGACTGAGCGACTTCACTTTCACTTTTCCCTTTCATG

CACTGGAGAAGGAAATGGCAACCCACTCCAGTATTCTTGCCTGGAGAATCCTAGGGACGGCGGAGCCTGG

TGGGCTGCCATCTATGGGGTCGCACAGAGTCGGACACGACTGAAGCGACTTAGCAGCAGCAGCAGCAGCA

GC

>Bov-A2.33.2

CGGAGAAGGCAATGGCACCCCACTCCAGTACTCTTGCCTGGAAAATCCCATGGACGGAGGAGCCTGGTAG

GCTACAGTCCATGGGGTCGCTAAGAGTCGGACACGACTGAGCGACTTCACTTTCACTTTTCCCTTTCATG

CACTGGAGAAAGAAATGGCAACCCACTCCAGTATTCTTGCCTGGAGAATCCTAGGGACGGCGGAGCCTGG

TGAGCTGCCGTCTATGGGGTCGCACAGAGTTGGACACGACTGAAGCGACTTAGCATAGCATAGCATAGCA

T

>Bov-A2.34.2

CGGAGAAGGCAATGGCACCCCACTCCAGTACTCTTGCCTGGAAAATCCCATGGACGGAGGAGCCTGGTAG

GCTGCAGTCCATGGGGTCGCTAAGAGTCGGACACGACTGAGCGACTTCACTTTCACTTTTCCCTTTCATG

CACTGGAGAAAGAAATGGCAACCCACTCCAGTATTCTTGCCTGGAGAATCCTAGGGACGGCAGAGCCTGG

TGAGCTGCCGTCTATGGGGTCGCACAGAGTCGGACACGACTGAAGCGACTTAGCATAGCAT

>Bov-A2.35.2

CGGAGAAGGCAATGGCACCCCACTCCAGTACTCTTGCCTGGAAAATCCCATGGACGGAGGAGCCTGGTAG

GCTACAGTCCATGGGGTCGCTAAGAGTCGGACACGACTGAGCGACTTCACTTTCACTTTTCCCTTTCATG

CACTGGAGAAAGAAATGGCAACCCACTCCAGTATTCTTGCCTGGAGAATCCTAGGGACGGCAGAGCCTGG

TGAGCTGCCGTCTATGGGGTCGCACAGGGTCGGACACGACTGAAGCGACTTAGCATAGCATAGCAT

>Bov-A2.36.2

CGGAGAAGGCAATGGCACCCCACTCCAGTACTCTTGCCTGGAAAATCCCATGGACGGAGGAGCCTGGTAG

GCTACAGTCCATGGGGTCGCTAAGAGTCGGACACGACTGAGCGACTTCACTTTCACTTTTCCCTTTCATG

CACTGGAGAAAGAAATGGCAACCCACTCCAGTATTCTTGCCTGGAGAATCCTAGGGACGGCAGAGCCTGG

TGAGCTGCCGTCTATGGGGTCGCACAGAGTCGGACACAACTGAAGCGACTTAGCATAGCATAGCAT

>Bov-A2.37.2

CGGAGAAGGCAATGGCACCCCACTCCAGTACTCTTGCCTGGAAAATTCCATGGACGGAGGAGCCTAGTAG

GCTGCAGTCTATGGGGTCGCTAGGGGTCGGTCACGACTGAGCGGCTTCACTTTCACTTTTCACTTTCATG

CATTGGAGAAGGAAATGGCAGCCCACTCCAGTATTCTTGCCTGGAGAATCCCAGGGACGGGGGAGCCCAG

TGGGCTGCCGTCTATGGGGTCGCACAGAGTCGGACACGACTGAAGCGACTTAGCAGCAGCAGC

>Bov-A2.38.2

CGGAGAAGGCAATGGCACCCCACTCCAGTACTCTTGCCTGGAAAATCCCATGGACGGAGGAGCCTAGTAG

GCTACAGTCCATGGGGTCGCTAAGAGTCGGACACGACTGAGCGACTTCACTTTCACTTTACCCTTTTATG

CACTGGAGAAGGAAATGGCAACCCACTCCAGTATTCTTGCCTGGAGAATCCCAGGGACGGCGGAGCCTGG

TGGGCTGCCGTCTATGGGGTCGCACAGAGTCGGACACGACTGAAGCGACTTAGCAGCAGCAGCAGCAGCA

GC

>Bov-A2.39.2

CGGAGAAGGCAATGGCATCCCACTCCAGTACTCTTGCCTGGAAAATCCCATGGACGGAGGAGCCTGGTAG

GCTACAGTCCATGGGGTCGCTAAGAGTCGGACACGACTGAGCGACTTCACTTTCACTTTTCCCTTTCATG

CACTGGAGAAGGAAATGGCAACCCACTCCAGTATTCTTGCCTGGAGAATCCCAGGGACGGTGGAGCCTGG

TGGGCTGCCGTCTATGGGGTCGCACAGAGTCGGACACGACTGAAGCGACTTAGCAGTAGCAGCAGCAGC

>Bov-A2.40.2

CGGAGAAGGCAATGGCACCCCACTCCAGTACTCTTGCCTGGAAAATCCCATGGATGGAGGAGCCTGGTAG

GCTATATAGTCCATGGGGTCGCTAAGAGTCGGACACGACTGAGCGACTTCACTTTCACTTTTCCCTTTCA

TGCACTGGAGAAGGAAATGGCAACCCACTCCAGTATTCTTGCCTGGAGAATCCCAGGGACGGTGGAGCCA

GGTGGGCTGCCATCTGTGGGGTCGCACAGAGTCGGACACGACTGAAGCGACTTAGCAGCAGCAGCAGCAG

CAGC

>Bov-A2.41.2

CGGAGAAGGAAATGGCAACCCACTCCAGTACTCTTGCCTGGAAAATCCTATGGACAGAGGAGCCTGGTAG

GCTGCAGTCCGTGGGTTCGCTAAGGGTCGGACACGACTAAGCGACTTCACTTTCACTTTTCCCTTTCATG

CACTGGAGAAGGAAATGGCAACCCACTCCAGTATTCTTGCCTGGAGAATCCTAGGGACGGCAGAGCCTGG

TGGGCTGCCGTCTATAGGGTCGCACAGAGTCGGACACGACTGAAGCGACTTAGCAGTAGCAGCAGCAGC

>Bov-A2.42.2

CGGAGAAGGCAATGGCACCCCACTCCAGTACTCTTGCCTGGAAAATCCCATGGACGGAGGAGCCTGGTAG

GCTACAGTCCATGGGGTCGCTAAGAGTCGGACACGACTGAGCGACTTCACTTTCACTTTTCCCTTTCATG

CACTGGAGAAGGAACTGGCAACCCACTCCAGTATTCTTGCCTGGAGAATCCCAGGGACGGCGGAGCCTGG

TGGGCTGCCGTCTATGGGGTCGCACAGAGTCGGACACGACTGAAGCGACTTAGCAGCAGCAGCAGC

>Bov-A2.43.2

CGGAGAAGGCAATGGCACCCCACTCCAGTACTCTTGCCTGGAAAATCCCATGGACGGAGGAGCCTGGTAG

GCCACAGTCCATGGGGTCGCTAAGAGTCGGACACGACTGAGCGACTTCACTTTCACTTTTCCCTTTCATG

CACTGGAGAAGGAAATGGCAACCCACTCCAGTATTCTTGCCTGGAGAATCCCAGGGACGGCGGAGCCTGG

TGGGCTGCCGTCTATGGGGTCGCACAGAGTCGGACACGACTGAAGCGACTTAGCAGCAGCAGC

>Bov-A2.44.2

CGGAGAAGGCAATGGCACCCCACTCCAGTACTCTTGCCTGGAAAATCCCATGGACGGAGGAGCCTGGTAG

GCTACAGTCCATGGGGTCGCTAAGAGTCGGACACGACTGAGCGACTTCACTTTCACTTTTCCCTTTCATG

CACTGGAGAAGGAAATGGCAACCCACTCCAGTATTCTTGCCTGGAGAATCCCAGGGACGGTGGAGCCTGG

TGGGCTGCCGTCTATGGGGTCGCACAGAGTCGGACACGACTGAAGCGACTTAGCAGCAGCAGCAGC

>Bov-A2.45.2

CGGAGAAGGCAATGGCACCCCACTCCAGTACTCTTGCCTGGAGAATCCCATGGACGGAGGAGCCTGGTAG

GCTACAGTCCATGGGGTCGCTAAGAGTCGGACACGACTGAGCGACTTCACTTTCACTTTTCCCTTTCATG

CACTGGAGAAGGAAATGGCAACCCACTCCAGTATTCTTGCCTGGAGAATCCCAGGGACGGCGGAGCCTGG

TGGGCTGCCGTCTATGGGGTCGCACAGAGTCGGACACGACTGAAGCGACTTAGCAGCAGCAGCAGC

>Bov-A2.46.2

TGGAGAAGGCAATGGCACCCCACTCCAGTACTCTTGCCTGGAAAATCCCATGAACGGAGGAGCCTGGCGG

GCTGCAGTCCATGGGGTCACTGAGTCGGACACGACTGAGCGACTCCACTTTCACTTTTCACTTTCATGCC

CTGGAGAAGGAAATGGCAACCCACTCCAGTACTCTTGCCTGGAGAATCCCAGGGATGGGGGAGCCTGGTG

GGCTGCCGTCTATGGGGTCGCACAGAGTCGGACACGACTGACGCGACTTAGCAGCAGCAGCAGC

>Bov-A2.47.2

CGGAGAAGGCAATGGCACCCCACTCCAGTACTCTTGCCTGGAAAATCCCATGAACGGAGGAGCCTGGCGG

GCTGCAGTCCATGGGGTCACTGAGTCGGACACGACTGAGTGACTCCACTTTCACTTTTCACTTTCATGCC

CTGGAGAAGGAAATGGCAACCCACTCCAGTACTCTTGCCTGGAGAATCCCAGGGACGGGGGAGCCTGGTG

GGCTGCCGTCTATGGGGTCGCACAGAGTCGGACACGACTGACGCGACTTAGCAGCAGCAGCAGCAGC

>Bov-A2.48.2

CGGAGAAGGCAATGGCACCCCACTCCAGTACTCTTGCCTAGAAAATCCCATGGATGGAGGAGCCTGGTAG

GCTACAGTCCATGGGGTCGCTACGAGTCGGACACGACTGAGCAACTTCACTTTCACTTTTCCCTTTCATG

CACTGGAGAAGGAAATGGCAACCCACTCCAGTATTCTTGCCTGGAGAATCCTAGGGACGGCGGAGCCTGG

TGGGCTGCCGTCCATGGGGTCGCACAGAGTCGGACACGACTGAAGCGACTTAGCAGCAGCAGCAGC

>Bov-A2.49.2

CGGAGAAGGCAATGGCACCCCACTCCAGTACTCTTGCCTGGAAAATCCCATGGACAGAGGAGCCTGGTAG

GCTGCAGTCCATGGGGTCGCTAAGAGTCAGACATGACTGAGCAACTTCACTTTCCCTTTTCACTTTCATG

CATTGGAGAAGGAAATGGCAACCCACTCCAGTGTTCTTGCCTGGAGAATCCCAGCGACGGGGGAGCCTGG

TGGGCTGCCGTCTATGGGGTCGCACAGAGTCGGACACGACTGAAGCGACTTAGCACTTAGC

>Bov-A2.50.2

CGGAGAAGGCAATGGCACCCCACTCCAGTACTCTTGCCTGGAAAATCCCATGGACAGAGGAGCCTGGTAG

GCTGCAGTCCATGGGGTCGCTAAGAGTCGGACACGACTGAGCGACTTCACTTTCACTTTTCCCTTTCATG

CATTGGAGAAGGAAATGGCAACCCACTCCAGTGTTCTTGCCTGGAGAATCCCAGGGACAGCGGAGCCTGG

TGGGCTGCCGTCTATGGGGTCGCACAGAGTCGGACACGACTGAAGCGACTTAGCAGTAGCAGTAGCAGT

>Bov-A2.51.2

CGGAGAAGGCAATGGCACCCCACTCCAGTACTCTTGCCTGGAAAATCCCATGGATGGAGGAGCCTGGTAG

GCTGTAGTCCATGGGGTCGCTAAGAGTCGGACACGACTGAGCGACTTCACTTTCACTTTTCCCTTTCATG

CACTGGAGAAGGAAATGGCAACCCACTCCAGTATTCTTGCCTGGAGAATCCTAGGGACGGCGGAGCCTGG

TGGGCTGCCGTCTATAGGGTCGCACAGAGTCGGACACGACTGAAGCGACTTAGCAGCAGCAGC

>Bov-A2.52.2

CGGAGAAGGCAATGGCACCCCACTCCAGTACTCTTGCCTGGAAAATCCCATGGGTGGAGGAGCCTGGTAG

GCTGCAGACCCATGGGGTCGCTAGGAGTCGGACATGACTGAGCGACTTCACTTTCACTTTTCCCTTTCAT

GCACTGGAGAAGGAAATGGCAACCCACTCCAGTATTCTTGCCTGGAGAATCCCAGGGACGGCGGAGCCTG

GTGGGCTGCCGTCTATGGGGTCGCACAGAGTCGGACACGACTGAAGCGACTTAGCAGCAGCA

>Bov-A2.53.2

TGGAGAAGGCAATGGCACCCCACTCCAGTACTCTTGCCTGGAAAATCCCATGAACGGAGGAGCCTGGCGG

GCTGCAGTCCATGGGGTCACTGAGTCGGACACGACTGAGCGACTCCACTTTCACTTTTCACTTTCATGCC

CTGGAGAAGGAAATGGCAACCCACTCCAGTACTCTTGCCTGGAGAATCCCAGGGACGGGGGAGCCTGGTG

GGCTGCCGTCTATGGGGTCGCACAGAGTCGGACACGACTGACACGACTTAGCAGCAGCAGCAGC

>Bov-A2.54.2

TGGAGAAGGCAATGGCACCCCACTCCAGTACTCCTGCCTGGAAAATCCCATGGATGGAGGAGCCTGGTAG

GCTACAGTCCATGGGGTCGCTAAGAGTCGGACACGACTGAGCGACTTCACTTTCACTTTTCCCTTTCATG

CACTGGAGAAGGAAATGGCAACCCACTCCAGTATTCTTGCCTGGAGAATCCCAGGGACGGCGGAGCCTGG

TGGGCTGCCGTCTATGGGGTCGCACAGAGTCGGACACGACTGAAGCGACTTAGCAGCAGCAGCAGCAGCA

>Bov-A2.55.2

CGGAGAAGGCAATGGCACCCCACTCCAGTACTCTTGCCTGGAAAATCCCATGGACGGAGGAGCCTGGTAG

GCTATAGTCCATGGGGTCGCTAAGAGTCGGACACGACTGAGCGACTTCACTTTAACTTTTCCCTTTCATG

CACTGGAGAAGGAAATGGCAACCCACTCCAGTATTCTTGCCTGGAGAATCCTAGGGACGGCGGAGCCTGG

TGGGCTGCCGTCTATAGGGTCGCACAGAGTCGGACACGACTGAAGCGACTTAGCAGCAGCAGCAGCAGC

>Bov-A2.56.2

CGGAGAAGGCAATGGCACCCCACTCCAGTACTCTTGCCTGGAAAATCCCATGGGTGGAGGAGCCTGGTAG

GCTGCAGACCCATGGGGTCGCTAGGAGTCGGACATGACTGAGCGACTTCACTTTCACTTTTCCCTTTCAT

GCACTGGAGAAGGAAATGGCAACCCACTCCAGTATTCTTGCCTGGAGAATCCCAGGGACGGCGGAGCCTG

GTGGGCTGCCGTCTATGGGGTCGCACAGAGTCGGACACGACTGAAGCGACTTAGCAGTAGCAGT

>Bov-A2.57.2

CGGAGAAGGCAACGGCACCCCACTCCAGTACTCTTGCCTGGAAAATCCCATGGATGGAGGAGCCTGGTAG

GCTACAGTCCATGGGGTCGCTGAGAGTCGGACACGACTGAGCGACTTCACTTTCACTTTTCCCTTTCATG

CACTGGAGAAGGAAATGGCAACCCACTCCAGTATTCTTGCCTGGAGAATCCCAGGGATGGCGGAGCTTGA

TGGGCTGCCGTCTATGGGGTCGCACAGAGTCGGACACGACTGAAGCGACTTAGCAGCAGCAGCAGCAGCA

GCAGC

>Bov-A2.58.2

CGGAGAAGGCAATGGCACCCCACTCCAGTACTCTTGCCTGGAAAATCCCAAGGACAGAGGAGCCTGGTAG

GCTACAGTCCATGGGGTCGCTAGAAGTCGGACACGACTGAGCAACTTCACTTCCACTTTTCCCTTTCACG

CACTGGAGAAGGAAATGGCAACCCACTCCAGTATTCTTGCCTGGAGAATCCCAGGGATGGGGGAGCCTGG

TGGGCTGCCATCTATGGGGTCGCACAGAGTCGGACACGACTGAAGCGACTTAGCAGCAGCAGCAGCAGC

>Bov-A2.59.2

CGGAGAAGGCAATGGCACCCCACTCCAGTACTCTTGCCTGGAAAATCCCATGGACGGAGGAGCCTGGTAG

GCTACAGTCCATGGGGTCGCTAAGAGTCAGACACGACTGAGCGACTTCACTTTCACTTTTCCCTTTCATG

CACTGGAGAAAGAAATGGCAACCCACTCCAGTATTCTTGCCTGGAGAATCCTAGGGACGGCGGAGCCTGG

TGAGCTGCCGTCTATGGGGTCGCACAGAGTCGGACACGACTGAAGCGACTTAGCATAGCATAGCATAGCA

T

>Bov-A2.60.2

CGGAGAAGGTAATGGCACCCCACTCCAGTACTCTTGCCTGGAAAATCCCATGGACGGAGGAGCCTCGTAG

GCTGCAGTCCATGGGGTCGCTAAGAGTCGGACACGACTGAGCGATTTCACTTTCACTTTTCACTTTCATG

CATTGGAGAAGGAAATGGCAACCCACTCCAGTGTTCTTGCCTGGAGAATCCCAGGGACGGCGGAGCCTGG

TGGGCTGCCGTCTATGGGGTCGCACAGAGTCGGACACGACTGAAGCGACTTAGCAGCAGCAGCAGCAGC

>Bov-A2.61.2

CGGAGAAGGCAATGGCACCCCACTCCAGTACTCTTGCCTGGAAAATCCCATGGACGGAGGAGCCTGGTAG

GCTACAGTCCATGGGGTCGCTAAGAGTCGGACACGACTGAGCGACTTCACTTTCACTTTTCCCTTTCATG

CACTGGAGAAGGAAATGGCAATCCACTCCAGTATTCTTGCCTGGAGAATCCCAGGGACGGCGGAGCCTGA

TGGGCTGCCGTCTATGGGGTCGCACAGAGTCGGACACGACTGAAGCGACTTAGCAGCAGCAGCAGCAGC

>Bov-A2.62.2

CGGAGAAGGCAATGGCACCCCACTCCAGTACTCTTGCCTGGAAAATCCCATGGATGGAGGAGCCTGGTAG

GCTACAGTCCATGGGGTCGCTAAGAGTCGGACACGACTGAGCGACTTCACTTTCACTTTTCCCTTTCATG

CACTGGAGAAGGAAATGGCAACCCACTCCAGTATTCTTGCCTGGAGAATCCCAGGGACGGCGGAGCCTGG

TGAGCTGCCGTCTATGGGGTCGCACAGAGTCGGACACGACTGAAGCGACTTAGCAGCAGCAGCAGC

>Bov-A2.63.2

CGGAGAAGGCAATGGCACCCCACTCCAGTACTCTTGCCTGGAAAATCCCATGGATGGAGGAGCCTGGTAG

GCTACAGTCCATGGGGTCGCTAAGAGTCGGACACGACTGAGCGACTTCACTTTCACTTTTCCCTTTCATG

CACTGGAGAAGGAACTGGCAACCCACTCCAGTATTCTTGCCTGGAGAATCCCAGGGACGGCGGAGCCTGG

TGGGCTGCCGTCTATGGGGTCGCACAGAGTCGGACACGACTGAAGCGACTTAGCAGCAGCAGCAGCAGCA

GC

>Bov-A2.64.2

CGGAGAAGGCAATGGCACCCCACTCCAGTACTCTTGCCTGGAAAATCCCATGGACGGAGGAGCCTGGTAG

GCTACAGTCCATGGGGTCGCTAAGAGTCGGACGACTGAGCGACTTCACTTTCACTTTTCCCTTTCATGCA

CTGGAGAAGGAAATGGCAACCCACTCCAGTATTCTTGCCTGGAGAATCCCAGGGACGGCGGAGCCTGGTG

GGCTGCCGTCTATGGGGTCGCACAGAGTCGGACACGACTGAAGCGACTTAGCAGCAGCAGCAGCAGC

>Bov-A2.65.2

CGGAGAAGGCAATGGCACCCCACTCCAGTACTCTTGCCTGGAAAATCCCATGGACGGAGGAGCCTGGTAG

GCTACAGTCCATGGGGTCGCTGAGAGTCGGACACGACTGAGCGACTTCACTTTCACTTTTCCCTTTCATG

CACTGGAGAAGGAAATGGCAACCCACTCCAGTATTCTTGCCTGGAGAATCCCAGGGATGGCGGAGCCTGG

TGGGCTGCCGTCTATGGGGTCGCACAGAGTCGGACACGACTGAAGCGACTTAGCAGCAGCAGCAGCAGCA

ACAGC

>Bov-A2.66.2

CGGAGAAGGCAATGGCACCCCACTCCAGTACTCTTGCCTGGAAAATCCCATGGATGGAGGAGCCTGGTAG

GCTACAGTCCATGGGGTCGCTAAGAGTCGGACACGACTGAGCGACTTCACTTTCACTTTTCCCTTTCATG

CACTGGAGAAGGAAATGGCAACCCACTCCAGTATTCTTGCCTGGAGAATCCCAGGGATGGCGGAGCCTGG

TGGGCTGCCGTCTATGGGGTCGCACAGAGTCGGACACGACTGAAGCGACTTAGCAGCAGCAGC

>Bov-A2.67.2

CGGAGAAGGCAATGGCACCCCACTCCAGTACTCTTGCCTGGAAAATCCCATGGACGGAGGAGCCTGGTAG

GCTGCAGTCCATGGGGTCGCTAAGAGTCGGACACGACTGAGCGACTTCACTTTCACTTTTCCCTTTCATG

CACTGGAGAAGGAAATGGCAACCCACTCCAGTATTCTTGCCTGGAGAATCCCAGGGACGGCGGAGCCTGG

TGGCTGCCGTCTATGGGGTCGCACAGAGTCGGACACGACTGAAGCGACTTAGCAGCAGCAGCAGCAGCAG

C

>Bov-A2.68.2

TGGAGAAGGCAATGGCACCCCACTCCAGTACTCTTGCCTGGAAAATCCCATGGACGGAGGAGCCTGGTAG

GCTACAGTCCATGGGGTCGCTAAGAGTCGGACACGACTGAGCGACTTCACTTTCACTTTTCCCTTTCATG

CACTGGAGAAAGAAATGGCAACCCACTCCAGTATTCTTGCCTGGAGAATCCTAGGGACGGCGGAGCCTGG

TGGGCTGCCGTCTATGGGGTCGCACAGAGTCGGACACGACTGAAGCGACTTAGCATAGCATAGCATAGCA

T

>Bov-A2.69.2

CGGAGAAGGCAATGGCACCCCACTCCAGTACTCTTGCCTGGAAAATCCCATGGATGGAGGAGCCTGGTAG

GCTACAGTCCATGGGGTCGCTGAGAGTCGGACACGACTGAGCGACTTCACTTTCATTTTTCCCTTTCATG

CACTGGAGAAGGAAATGGCAACCCACTCCAGTATTCTTGCCTGGAGAATCCCAGGGATGGCGGAGCCTGG

TGGGCTGCCGTCTATGGGGTCGCACAGAGTCGGACACGACTGAAGCGACTTAGCAGCAGCAGCAGC

>Bov-A2.70.2

CGGAGAAGGCAATGGCACCCCACTCCAGTACTCTTGCCTGGAAAATCCCATGGATGGAGGAGCCTGGTAG

GCTACAGTCCGTGGGGTCGCTAAGAGTCGGACACGACTGAGCGACTTCACTTTCACTTTTCCCTTTCATG

CACTGGAGAAGGAAATGGCAACCCACTCCAGTATTCTTGCCTGGAGAATCCCAGGGATGGCAGAGCCTGG

TGGGCTGCCGTCTATGGGGTCGCACAGAGTCGGACACGACTGAAGCGACTTAGCAGCAGCAGCAGC

>Bov-A2.71.2

CGGAGAAGGCAATGGCACCCCACTCCAGTACTCTTGCCTGGAAAATCCCATGGATGGAGGAGCCTGGTAG

GCTACAGTCCATGGGGTCGCTAAGAGTCGGACACGACTGAGCGACTTCACTTTCACTTTTCCCTTTCATG

CACTGGAGAAAGAAATGGCAACCCACTCCAGTATTCTTGCCTGGAGAATCCTAGGGACGGCAGAGCCTGG

TGAGCTGCCGTCTATGGGGTCGCACAGAGTCGGACACGACTGAAGCGACTTAGCATAGCATAGCATAGCA

T

>Bov-A2.72.2

CGGAGAAGGCAATGGCACCCCACTCCAGTACTCTTGCCTGGAAAATCCCATGGACGGAGGAGCCTGGTAG

GCTACAGTCCATGGGGTCGCTAAGAGTCGGACGACTGAGCGACTTCACTTTCACTTTTCCCTTTCATGTA

CTGGAGAAGGAAACGGCAACCCACTCCAGTATTTTTGCCTGGAGAACCCCAGGGACGGCGGAGCCTGGTG

GGCTGCCGTCTATGGGGTCGCACAGAGTCGGACACGACTGAAGCGACTCAGCAGCAGCAGCAGCAGC

>Bov-A2.73.2

CGGAGAAGGCAATGGCACCCCACTCCAGTACTCTTGCCTGGAAAATCCCATGGACGGAGGAGCCTGGTAG

GCTACAGTCCATGGGGTTGCTAAGAGTCGGACGACTGAGCGACTTCACTTTCACTTTTCCCTTTCATGCA

CTGGAGAAGGAAACGGCAACCCACTCCAGTATTCTTGCCTGGAGAATCCCAGGGACGGCGGAGCCTGGTG

GGCTGCCGTCTATGGGGTCGCACAGAGTCGGACACGACTGAAGCGACTTAGCAGCAGT

>Bov-A2.74.2

GGAGAAGGCAATAGCAACCCACTCCAGTACTCTTGCCTGGAAAATCCCATGGACAGAGGAGCCTGGTAGG

CTGCAGTCCATGGGGTCGCGAAGAGTCGGACCCGACTGAGCGGCTTCACTTTTACTTTTCACTTTCATGC

ATTGGAGAAGGAAATGGCAACCCACTCCAGTGTTCTTGCCTGGAGAATCCCAGGGACAGGGGAGCCTGGT

GGGCTGCCGTCTATGGGGTCGCACAGAGTCGGACACGACTGAAGCGACTTAGCAGCAGCAGCAGC

>Bov-A2.75.2

CGGAGAAGGCGATGGCACCCCACTCCAGTACTCTTGCCTGGAAAATCCCATGGATGGAGGAGCCTGGTAG

GCTACAGTCCATGGGGTCGCTGAGTCGGACACGACTGAGCAACTTCACTTTCACTTTTCCCTTTCATGCA

CTGGAGAAGGAAATGGCAACCCACTCCAGTATTCTTGCCTGGAGAATCCCAGGGATGGCGGAGCCTGTGG

GTTGCCATCTATGGGGTCGCACAGAGTCGGACACGACTGAAGTGACTTAGCAGCAGCAGCAGCAGCAGC

>Bov-A2.76.2

CGGAGAAGGCAATGGCACCCCACTCCAGTACTCTTGCCTGGAAAATCCCATGGACGGAGGAGCCTGGTAG

GCTACAGTCCATGGGGTCGCTAAGAGTCGGACACGACTGAGCGACTTCACTTTCACTTTTCACTCTCATG

CATTGGAGAAGGAAATGGCAACCCACTCCAGTATTCTCGCCTGGAGAATCCTAGGGACGGCGGAGCCTGG

TGAGCTGCCGTCTATGGGGTCGCACAGAGTCGGACACGACTGAAGCGACTTAGCATAGCATAGCAT

>Bov-A2.77.2

CGGAGAAGGCAATGGCAACCCACTCCAGTACTCTTGCCTGGAAAATCCCATGGACGGAGGAGCCTGGTAG

GCTGCAGTCCATGGGGTCGCTAAGAGTCGGACACGACTGAGCAACTTCACTTTCACTTTTCACTTTCATG

CATTGGAGAAGGCAATGGCAACCCACTCCAGTGTTCTTGCCTGGAGAATCCCAGGGATGGGGGAGCCTGG

TGGGCTACCGTCTATGGGGTCGCACAGAGTCGGACACGACTGAAGCGACTTAGCAGCAGCAGCAGC

>Bov-A2.78.2

CGGAGAAGGCAATGGCACCCCACTCCAGTACTCTTGCCTGGAAAATCCCATGGACGGAGGAGCCTAGTAG

GCTACAGTCCATGGGGTCGCTAAGAGTCGGACACGACTGAGCGACTTCACTTTCACTTTACCCTTTCATG

CACTGGAGAAGGAAATGGCAACCCACTCCAGTATTCTTGCCTGGAGAATCCCAGGGACGGCGGAGCCTGG

TGGGCTGCCGTCTATGGGGTCGCACAGAGTCGGACACGACTGAAGCAACTTAGCAGCAGCAGC

>Bov-A2.79.2

CGGAGAAGGCAATGGCACCCCACTCCAGTACTCTTGCCTGGAAAATCCCATGGATGGAGGAGCCTGGTAG

GCTACAGTCCATGGGGTCGCTAAGAGTCGGACACGACTGAGCGACTTCACTTTCACTTTTCCCTTTCATG

CACTGGAGAAGGAAATGGCAACCCACTCCAGTATTCTTGCCTGGAGAATCCCAGGGACGGCAGAGCCTGG

TGGGCTGCCGTCTATGGGGTCGCACAGAGTCAGACACGACTGAAGCGACTTAGCAGCAGCAGCAGCAGCA

GC

>Bov-A2.80.2

CAGAGAAGGCAATGGCACCCCACTCCAGTACTCTTGCCTAGAAAATCCCATGGATGGAGGAGCCTGGTAG

GCTGCAGTCCATGGGGTCGCTAAGAGTCGGACACGACTGAGCGACTTCACTTTCACTTTTCCCTTTCATG

CACTGGAGAAGGAAATGGCAACCCACTCCAGTATTCTTGCCTGGAGAATCCCAGGGACGGCGGAGCCTGG

TGGGCTGCCGTCTATGGGGTCGCACAGAGTCGGACACGACTGAAGCGACTTAGCATAGCATAGCATAGCA

T

>Bov-A2.81.2

CGGAGAAGGCAATGGCACCCCACTCCAGTACTCTTGCCTGGAAAATCCCATGGACGGAGGAGCCTAGTAG

GCTGCAGTCTATGGGGTCGCTAGGGGTCAGACACGACTGAGCGGCTTCACTTTCACTTTTCACTTTCATG

CATTGGAGAAGGAAATGGCAGCCCACTCCAGTATTCTTGCCTGGAGAATCCCAGGGACGGGGGAGCCCGG

TGGGCTGCCGTCTATGGGGTCGCACAGAGTCGGACACGACTGAAGCGACTTAGCAGCAGCAGCAGCAGCA

GC

>Bov-A2.82.2

CGGAGAAGGCAATGGCACCCCACTCCAGTACTCTTGCCTGGAAAATCCCATGGACGGAGGAGCCTGGTAG

GCTACAGTCCATGGGGTCGCTAAGAGTCGGACACGACTGAGCGACTTCACTTTCACTTTTCCCTTTCATG

CACTGGAGAAGGAAATGGCAACCCACTCCAGTATTCTTGCCTGGAGAATCCTAGGGACGGCGGAGCCTGG

TGAGCTGCCGTCTATGGGGTCGCACAGAGTCGGACACGACTGAAGCGACTTAGCATAGCATAGCAT

>Bov-A2.83.2

CGGAGAAGGCAATGGCACCCCACTCCAGTACTCTTGCCTGGAAAATCCCATGGACGGAGGAGCCTGGTAG

GCTGTAGTCCATGGGGTTGCTAAGAGTCAGACATGACTGAGCGACTTCACTTTCACTTTTCCTGTTCATG

CACTGGAGAAGGAAATGGCAACCCACTCCAGTATTCTTGCCTGGAGAATCCCAGGGATGGCGGAGCCTGG

TGGGCTGCCGTCTATGGGGTCGCACAGAGTCGGACACGACTGAAGCGACTTAGCAGCAGCAGCAGCAGC

>Bov-A2.84.2

CGGAGAAGGCAATGGCACCCCACTCCAGTACTCTTGCCTGGAAAATCCCATGGACGGAGGAGCCTGGTAG

GCTGTAGTCCATGGGGTCGCTAAGAGTCAGACATGACTGAGCGACTTCACTTTCACTTTTCCTGTTCATG

CACTGGAGAAGGAAATGGCAACCCACTCCAGTATTCTTGCCTGGAGAATCCCAGGGATGGCGGAGCCTGG

TGGGCTGCCATCTATGGGGTCGCACAGAGTCGGACACGACTGAAGCGACTTAGCAGCAGCAGCAGCAGCA

GCAGC

>Bov-A2.85.2

CGGAGAAGGCAATGGCACCCCACTCCAGTACTCTTGCCTGGAAAATCCCATGGACGGAGGAGCCTGGTAG

GCTACAGTCCATGGGATCGCTAAGAGTCAGACACGACTGAGCGACTTCACTTTCACTTTTCCCTTTCATG

CACTGGAGAAAGAAATGGCAACCCACTCCAGTATTCTTGCCTGGAGAATCCTAGGGACGGCAGAGCCTGG

TGAGCTGCCGTCTATGGGGTCGCACAGAGTCGGACACGACTGAAGCGACTTAGCATAGCAT

**Sequences of 23 Bov-tA elements representing 23 clusters composed of identical Bov-tA copies, where Bov-tA.N1.N2 stays for the Bov-tA cluster number N1, which is composed of N2 identical sequences (all segmental duplications have been excluded):**

>Bov-tA.1.11

GGACTTCCCTGGTGGCTCAGAGGTTAAAGCGTCTGCCTCCAATGCGGGAGACCTGGGTTCGATCCCTGGG

TCGGGAAGATCCCCTGGAGAAGGAAATGGCAAACCACTCCAGTATTCTTGCCTGGAGAATCCCATGGACG

GAGGAGCCTGGCAGGCTACAGTCCACGGGGTCGCAAAGAGTCGGACACGACTGAGCGACTTTCCTTTC

>Bov-tA.2.6

GGACTTCCCTGGTGGCTCAGAGGTTAAAGCGTCTGCCTCCAATGCGGGAGACCTGGGTTCGATCCCTGGG

TCGGGAAGATCCCCTGGAGAAGGAAATGGCAAACCACTCCAGTATTCTTGCCTGGAGAATCCCATGGACG

GAGGAGCCTGGTAGGCTACAGTCCACGGGGTCGCAAAGAGTCGGACACGACTGAGCGACTTCCCTTCC

>Bov-tA.3.5

GACTTCCCTGGTGGCTCAGAGGTTAAAGCGTCTGCCTCCAATGCGGGAGACCTGGGTTCGATCCCTGGGT

CGGGAAGATCCCCTGGAGAAGGAAATGGCAAACCACTCCAGTATTCTTGCCTGGAGAATCCCATGGACGG

AGGAGCCTGGTAGGCTACAGTCCACGGGGTCGCAAAGAGTCGGACACGACTGAGCGACTTCCCTTCC

>Bov-tA.4.4

GGACTTCCCTGGTGGCTCAGAGGTTAAAGCGTCTGCCTCCAATGCGGGAGACCTGGGTTCGATCCCTGGG

TCAGGAAGATCCCCTGGAGAAGGAAATGGCAAACCACTCCAGTATTCTTGCCTGGAGAATCCCATGGACG

GAGGAGCCTGGTAGGCTACAGTCCACGGGGTCGCAAAGAGTCGGACACGACTGAGCGACTTCCCTTTCCT

>Bov-tA.5.4

GACTTCCCTGGTGGCTCAGAGGTTAAAGCGTCTGCCTCCAATGCGGGAGACCTGGGTTCGATCCCTGGGT

CGGGAAGATCCCCTGGAGAAGGAAATGGCAAACCACTCCAGTATTCTTGCCTGGAGAATCCCATGGACGG

AGGAGCCTGGTAGGCTACAGTCCATGGGGTCGCAAAGAGTCGGACACGACTGAGCGACTTCCCTTCCCTT

CC

>Bov-tA.6.4

GGACTTCCCTGGTGGCTCAGAGGTTAAAGCGTCTGCCTCCAATGCGGGAGACCTGGGTTCGATCCCTGGG

TCGGGAAGATCCCCTGGAGAAGGAAATGGCAAACCACTCCAGTGTTCTTGCCTGGAGAATCCCATGGACG

GAGGAGCCTGGCAGGCTACAGTCCACGGGGTCGCAAAGAGTCGGACACGACTGAGCGACTTTCCTTTC

>Bov-tA.7.3

GGACTTCCCTGGTGGCTCAGAGGTTAAAGCGTCTGCCTCCAATGTGGGAGACCTGGGTTCGATCCCTGGG

TCGGGAAGATCCCCTGGAGAAGGAAATGGCAAACCACTCCAGTGTTCTTGCCTGGAGAATCCCATGGACG

GAGGAGCCTGGCAGGCTACAGTCCACGGGGTCGCAAAGAGTCGGACACGACTGAGCGACTTTCCTTTCCT

TTC

>Bov-tA.8.3

GGACTTCCCTGGTGGCTCAGAGGTTAAAGCGTCTGCCTCCAATGCGGGAGACCTGGGTTCGATCCCTGGG

TCGGGAAGATCCCCTGGAGAAGGAAATGGCAAACCACTCCAGTATTCTTGCCTGGAGAATCCCATGGACG

GAGGAGCCTGGTAGGCTACAGTCCACGGGGTCGCAAAGAGTCGGACACGACTGAGCGACTTCCCTTTCCC

TT

>Bov-tA.9.3

GGACTTCCCTGGTGGCTCAGAGGTTAAAGCGTCTGCCTCCAATGCGGGAGACCTGGGTTCGATCCCTGGG

TCGGGAAGATCCCCTGGAGAAGGAAATGGCAAACCACTCCAGTATTCTTGCCTGGAGAATCCCATGGACG

GAGGAGCCTGGTAGGCTACAGTCCATGGGGTCGCAAAGAGTCGGACACGACTGAGCGACTTCCCTTCCCT

TCC

>Bov-tA.10.2

GGACTTCCCTGGTGGCTCAGAGGTTAAAGCGTCTGCCTCCAATGCGGGAGACCTGGGTTCGATCCCTGGG

TCGGGAAGATCCTCTGGAGAAGGAAATGGCAAACCACTCCGGTATTCTTGCCTGGAGAATCCCATGGACG

GAGGAGCCTGGTAGGCTACGGTCCACGGGGTCGCAAAGAGTCGGACACGACTGAGCGACTTCCCTTCC

>Bov-tA.11.2

CCTTAGTCCTGGTGGCTCAGATGGTAAAGCGACTGTCTACAATGCGGGAGACCTGGGTTCGATCCCTGGG

TTGGGAGGATCCCTGGAGAAGGAAATGGCAATCCACTCCAGTACTCTTGCCTAGAAAATCCCATGGACGG

AGGAGCCTGGTGTCCATGGGGTCACAAAGAGTCGGACACGACTGAGAGACTTCACTTTCACTTT

>Bov-tA.12.2

GAGCTTCCCTGGTGGCTCAGAGGTTAAAGCGTCTGCCTCCAATGTGGGAGACCTGGGTTCAATCCCTGGG

TCGGGAAGATCTCCTGGAGAAGGAAATGGCAACCCACTCCAGTATTCTTGCCTGGAGAATCCCATGGATG

GAGGATCCTGGTGGGCTACAGTCCATGGGGTCGCAAAGAGTCGGACACGACTGAGCGACTTCACTTCACT

TCAC

>Bov-tA.13.2

GACTTCCCTGGTGGCTCAGAGGTTAAAGCGTCTGCCTCCGATGCAGGAGACCTGGGTTCGATCCCTGGGT

TGGGAAGATCCCCTGGAGAAGGAAATGGCAACCCACTCCAGTATTCTTGCCTGGAGAATCCCATGGACGG

AGGAGCCTGGTAGGCTACAGTCCATGGGGTCGCAAAGAGTCGGACACGACTGAGCGACTTCACTTTCACT

TTCACTT

>Bov-tA.14.2

GGACTTCCCTGGTGGCTCAGAGGTTAAAGCGTCTGCCTCCAATGCAGGAGACCTGGGTTCGATCCCTGGG

TCGGGAAGTTCCCCTGGAGAAGGAAATGGCAACCCACTCCAGTATTCTTGCCTGGAGAATCCCATGGACG

GAGGAGCCTGGTAGGCTACAGTCCACGGGGTCGCAAAGAGTCGGACACGACTGAGCGACTTCCCAGT

>Bov-tA.15.2

GGACTTCCCTGGTGGCTCAGAGGTTAAAGCGTCTGCCTCCAATGCGGGAGACCTGGGTTCGATCCCTGGG

TCGGGAAGATCCCCTGGAGAAGGAAATGGCAAACCACTCCAGTATTCTTGCCTGGAGAATCCCATGGACG

GAGGAGCCTGGCAGGCTACAGTCCACGGGGTTGCAAAGAGTCGGACACGACTGAGCGACTTTCCTTTCCT

TT

>Bov-tA.16.2

GACTTCCCTGGTGGCTCAGAGGTTAAAGCGTCTGCCTCCAATGCAGGAGACCTGGGTTCGATCCCTGGGT

CGGGAAGATCCCCTGGAGAAGGAAATGGCAAACCACTCCAGTGTTCTTGCCTGGAGAATCCCATGGACGG

AGGAGCCTGGCAGGCTACAGTCCACGGGGTCGCAAAGAGTCGGACACGACTGAGCGACTTTCCTTTCCTT

TCCTTT

>Bov-tA.17.2

GGAGCCCCTGGTGGCTCAGATGGTAAAGAGTCTGCCTGCAGTGCAGGAGACCTGGGTTCAATCCTTGGGT

CAGGAAGATCCCCTGGAGAAGGAAATGGCAACCCACTCCAGTATTCTTGCCTGGAGAATCCCATGGATGG

AGGAGCCTGGCAGGTTACAGTCCATGGGGTCACAAAGAGTCGGACACGACTGAGTGACTTCACTTCACTT

C

>Bov-tA.18.2

GGACTTCCCTGGTGGCTCAGAGGTTAAAGCGTCTGCCTCCAATGCGGGACACCTGGGTTCGATCCCTGGG

TCGGGAAGATCCCCTGGAGAAGGAAATGGCAAACCACTCCAGTATTCTTGCCTGGAGAATCCCATGGACG

GAGGAGCCTGGTAGGCTACAGTCCATGGGGTCGCAAAGAGTCGGACACGACTGAGCGACTTCCCTTCCCT

TCC

>Bov-tA.19.2

GACTTCCCTGGTGGCTCAGAGGTTAAAGCGTCTGCCTCCAATGCAGGAGACCCGGGTTCGATCCCTGGGT

CGGGAAGATCCCCTGGAGAAGGAAATGGCAACCCACTCCAGTATTCTTGCCTGGAGAATCCCATGGACGG

AGGAGCCTGGTAGGCTATAGTCCATGGGGTCGCAAAGAGTCGGACACGACTGAGCGACTTCACTCACTCA

CTC

>Bov-tA.20.2

GACTTCCCTGGTGGCTCAGAGGTTAAAGCGTCTGCCTCCAATGCGGGAGACCTGGGTTCGATCCCTGGGT

CGGGAAGATCCCCTGGAGAAGGAAATGGCAAACCACTCCAGTATTCTTGCCTGGAGAATCCCATGGACGG

AGGAGCCTGGTAGGCTACAGTCCACGGGGTCGCAAAGAGTCGGACACGACTGAGCGACTTCCCTTTCCTT

T

>Bov-tA.21.2

GGACTTCCCTGGTGGCTCAGAGGTTAAAGCGTCTGCCTCCAATGCGGGAGACCTGGGTTCGATCCCTGGG

TCGGGAAGATCCCCTGGAGAAGGAAATGGCAAACCACTCCAGTATTCTTGCCTGGAGAATCCCATGGACG

GAGGAGCCTGGCAGGCTACAGTCCACGGGGTCGCAAAGAGTCGGACACGACTGAGCGACTTCCCTTTCCC

TTT

>Bov-tA.22.2

GGACTTCCCTGGTGGCTCAGAGGTTAAAGCGTCTGCCTCCAATGCGGGAGACCTGGGTTCGATCCCTGGG

TCGGGAAGATCCCCTGGAGAAGGAAATGGCAAACCACTCCAGTATTCTTGCCTGGAGAATCCCATGGACA

GAGGAGCCTGGTAGGCTACAGTCCACGGGGTCGCAAAGAGTCGGACACGACTGAGCGACTTCCCTTCC

>Bov-tA.23.2

GGACTTCCCTGGTGGCTCAGAGGTTAAAGCGTCTGCCTCCAATGCGGGAGACCTGGGTTCGATCCCTGGG

TCGGGAAGATCCCCTGGAGAAGGAAATGGCAAACCACTCCAGTATTCTTGCCTGGAGAATCCCATGGACG

AAGGAGCCTGGTAGGCTACAGTCCACGGGGTCGCAAAGAGTCGGACACGACTGAGCGACTTCCCTTCCCT

TCC
